# LPA signaling acts as a cell-extrinsic mechanism to initiate cilia disassembly and promote neurogenesis

**DOI:** 10.1038/s41467-021-20986-y

**Published:** 2021-01-28

**Authors:** Huai-Bin Hu, Zeng-Qing Song, Guang-Ping Song, Sen Li, Hai-Qing Tu, Min Wu, Yu-Cheng Zhang, Jin-Feng Yuan, Ting-Ting Li, Pei-Yao Li, Yu-Ling Xu, Xiao-Lin Shen, Qiu-Ying Han, Ai-Ling Li, Tao Zhou, Jerold Chun, Xue-Min Zhang, Hui-Yan Li

**Affiliations:** 1 State Key Laboratory of Proteomics, National Center of Biomedical Analysis, Beijing, China; 2 Sanford Burnham Prebys Medical Discovery Institute, La Jolla, USA; 3 School of Basic Medical Sciences, Fudan University, Shanghai, China

**Keywords:** Cell signalling, Organelles

## Abstract

Dynamic assembly and disassembly of primary cilia controls embryonic development and tissue homeostasis. Dysregulation of ciliogenesis causes human developmental diseases termed ciliopathies. Cell-intrinsic regulatory mechanisms of cilia disassembly have been well-studied. The extracellular cues controlling cilia disassembly remain elusive, however. Here, we show that lysophosphatidic acid (LPA), a multifunctional bioactive phospholipid, acts as a physiological extracellular factor to initiate cilia disassembly and promote neurogenesis. Through systematic analysis of serum components, we identify a small molecular—LPA as the major driver of cilia disassembly. Genetic inactivation and pharmacological inhibition of LPA receptor 1 (LPAR1) abrogate cilia disassembly triggered by serum. The LPA-LPAR-G-protein pathway promotes the transcription and phosphorylation of cilia disassembly factors-Aurora A, through activating the transcription coactivators YAP/TAZ and calcium/CaM pathway, respectively. Deletion of Lpar1 in mice causes abnormally elongated cilia and decreased proliferation in neural progenitor cells, thereby resulting in defective neurogenesis. Collectively, our findings establish LPA as a physiological initiator of cilia disassembly and suggest targeting the metabolism of LPA and the LPA pathway as potential therapies for diseases with dysfunctional ciliogenesis.

## Abstract (web summary)

Dynamic assembly and disassembly of primary cilia is critical for tissue development and homeostasis. Here the authors identify lysophosphatidic acid (LPA) as a physiological extracellular factor that initiates cilia disassembly through both YAP/TAZ mediated transcription and calcium/calmodulin mediated activation of Aurora A.

## Introduction

The primary cilia are antenna-like organelles assembled on the surface of most mammalian cells^1,2^. They act as physical and chemical sensors and transducers of extracellular signals, and play critical roles in diverse cellular processes, including embryonic development and tissue homeostasis^3–6^. Cilia dysfunction is linked to tumorigenesis and numerous human developmental diseases collectively called ciliopathies^7–11^.

Ciliogenesis is dynamically controlled by cilia assembly and disassembly. Cilia assembly occurs in non-dividing (G0/G1) cells in response to serum deprivation, whereas cilia must be disassembled prior to cell cycle re-entry upon serum stimulation^12,13^. Many regulators of the cell cycle, such as Aurora kinase A (Aurora A), NIMA-related kinases (NEK) and Polo-like kinase 1 (PLK1), have emerged as crucial modulators of cilia disassembly^13–15^. Therefore, cilia disassembly is tightly coupled with cell cycle.

PDGF and IGF-1 at concentrations much higher than those in serum can induce cilia disassembly to a degree, but they do so much less efficiently than serum itself^13,16,17^. Thus, the major component in serum that induces cilia disassembly is unknown. Dynamic ciliogenesis is critical for tissue differentiation, including neurogenesis^18,19^. However, the extracellular cues controlling cilia disassembly in vivo remain elusive.

During cortical development in mammals, neurogenesis occurs within two germinal zones of the embryonic cerebral cortex—the ventricular zone (VZ) and the subventricular zone (SVZ)^20^. The neural progenitor cells (NPCs) from these two germinal zones generate projection neurons dedicated to all cortical layers^21,22^. The primary cilia in the radial glia (RG) cells in the VZ extend into the lateral ventricle and function as cellular antenna to detect signals present in the cerebrospinal fluid (CSF), thus regulating neurogenesis^23–25^. These cilia are highly dynamic. They are present during interphase and disassembled prior to entry into mitosis^26–28^. The mitotic entry of RG cells needs to be spatially and temporally regulated to coordinate their self-renewal divisions and asymmetric divisions to form the SVZ^20,29^. However, the developmental cues regulating cilia disassembly and mitotic entry of RG cells are unknown.

Lysophosphatidic acid (LPA), a multifunctional bioactive phospholipid, has been implicated in a variety of biological processes, including embryonic development and lymphocyte trafficking^30–35^. LPA is abundantly present in many tissues and in circulating blood^36^. It is mainly produced from lysophosphatidylcholine by the enzyme Autotaxin (Atx; *Enpp2*), a secreted lysophospholipase D (lysoPLD). LPA binds and activates LPA receptors (LPARs), an important class of G-protein-coupled receptors^37^. Upregulation of LPA signaling causes tumorigenesis^38^. *Lpar1*-depleted mice exhibited ~50% neonatal lethality and adult *Lpar1*^−/−^ survivors showing reduced size and craniofacial dysmorphism^32^.

In this study, we identify LPA as the major factor in serum for driving cilia disassembly. LPAR1 is critical for proper ciliogenesis, VZ/SVZ generation, and neocortex formation in mice. Our findings establish LPA as an important developmental cue of cilia disassembly, link defective ciliogenesis to diseases with elevated LPA signaling, and suggest chemical modulators of the LPA metabolism and LPA pathway as potential therapeutic agents for diseases with dysfunctional ciliogenesis.

## Results

### LPA is the major serum factor that triggers cilia disassembly

**Fig. 1:**
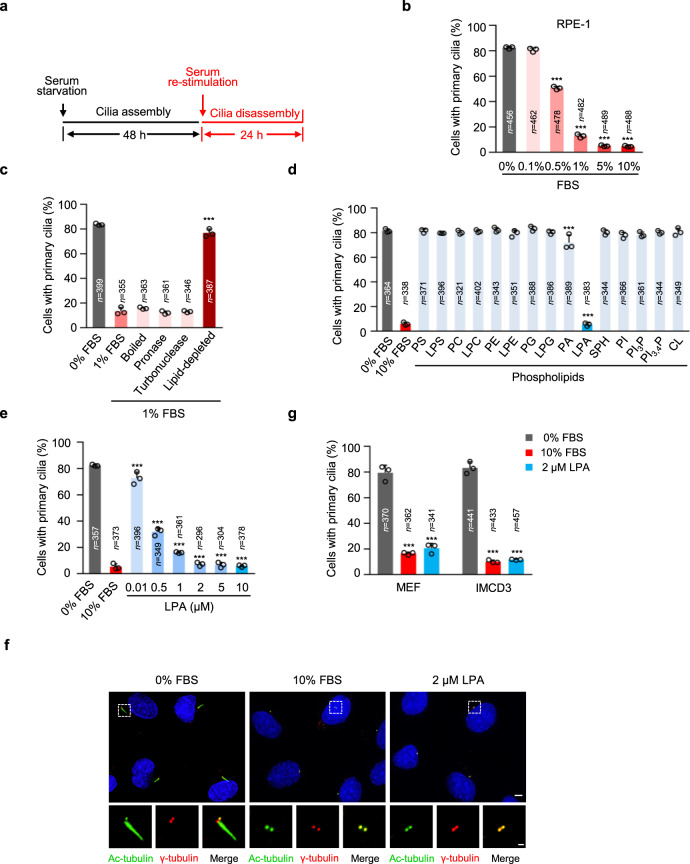
Identification of LPA as the major cilia disassembly factor in serum. **a** Diagram indicates the timing of serum starvation (cilia assembly) and re-stimulation (cilia disassembly). **b** Serum induces cilia disassembly in a dose-dependent manner. Human retinal pigment epithelial (RPE-1) cells were starved in DMEM/F12 for 48 h to induce cilia formation, and then ciliated RPE-1 cells were re-stimulated with indicated concentrations of FBS for 24 h to induce cilia disassembly. **c** Lipids are responsible for serum-induced cilia disassembly. Ciliated RPE-1 cells (serum-starved) were stimulated with 1% boiled, pronase-pretreated, turbonuclease-pretreated or lipid-depleted serum for 24 h, respectively. **d** Lysophosphatidic acid (LPA) induces cilia disassembly. Ciliated RPE-1 cells (serum-starved) were stimulated with 100 μM of various phospholipids for 24 h. Full name of lipids used are shown in “Methods.” **e** Dose-dependent effects of LPA on cilia disassembly. Ciliated RPE-1 cells (serum-starved) were treated with different concentrations of LPA for 24 h. **f** Representative images of RPE-1 cells in (**e**). Cells were stained with anti-Ac-tubulin (green) and anti-γ-tubulin (red) antibodies. Scale bar, 5 μm (main image) and 1 μm (magnified region). Three experiments were repeated independently with similar results. **g** LPA treatment (2 μM for 24 h) induces cilia disassembly in primary MEF and IMCD3 cells. Source data are provided as a Source Data file. Data are presented as mean ± S.D. of three independent experiments in (**b**–**e**) and (**g**). *n*, number of cells. ***P < 0.001. One-way ANOVA test was performed followed by Dunnett’s multiple comparisons in **b**–**e**; two-way ANOVA test was performed in followed by Dunnett’s multiple comparisons in **g**.

To identify the component in serum that can induce cilia disassembly, we set up a cilia disassembly assay with cultured human retinal pigment epithelial (RPE-1) cells^13^. Briefly, we serum-starved RPE-1 cells for 48 h to induce cilia formation in the majority of cells. Ciliated cells were then re-stimulated with serum for 24 h to trigger the disassembly of cilia (Fig. 1a). The effect of serum on cilia disassembly was dose-dependent (Fig. 1b). The minimum concentration of serum that could efficiently induce cilia disassembly was about 1% (Fig. 1b and Supplementary Fig. 1a). Since growth factors such as PDGF (platelet-derived growth factor) and IGF-1 (insulin-like growth factor 1) have been reported to weakly induce ciliary disassembly^13,16,17^, we tested several major growth factors in serum, including PDGF, IGF, EGF and FGF, in cilia disassembly. As previously reported, only PDGF at high concentrations caused limited cilia disassembly (Supplementary Fig. 1b). Therefore, these growth factors are unlikely to be the major components in serum that trigger cilia disassembly.

To determine which component in serum is responsible for cilia disassembly, we next destroyed the protein and nucleic acid components in serum by boiling or treating with pronase or turbonuclease and then tested the cilia-disassembly activity of the treated serum. Interestingly, the cilia-disassembly activity was resistant to all these harsh treatments (Fig. 1c and Supplementary Fig. 1c). These results suggest that the cilia disassembly activity may not be caused by proteins or nucleic acids. We next removed lipids from serum and treated ciliated cells with this lipid-depleted serum. We found that lipid-depleted serum had a markedly decreased ability to induce cilia disassembly (Fig. 1c and Supplementary Fig. 1c). This observation suggests that the factor(s) triggering cilia disassembly in serum might be lipid molecules.

To identify which lipids in serum-induced cilia disassembly, we tested a series of phospholipids in our cilia disassembly assay. Among the phospholipids tested, only lysophosphatidic acid (LPA) strongly induced cilia disassembly in RPE-1 cells (Fig. 1d and Supplementary Fig. 1d). Various LPA isoforms with different lengths and degrees of saturation of the fatty acid tail could efficiently trigger cilia disassembly (Supplementary Fig. 1e). As 18:1-LPA is the most widely used isoform in the laboratory^37^, we used 18:1-LPA in all subsequent LPA treatment experiments. LPA is an abundant phospholipid in serum and its concentration in human serum is in the μM range^39,40^. At this concentration, LPA alone could induce substantial cilia disassembly as revealed by dose response analysis (Fig. 1e, f), suggesting that LPA in serum is adequate to induce cilia disassembly. Given that cilia disassembly occurred in two waves after serum stimulation (1–2 h and 18–24 h, respectively)^13^, we next examined the disassembly waves induced by LPA treatment. Similar to serum, LPA treatment obviously induced cilia disassembly at 2, 18, and 24 h (Supplementary Fig. 1f). In addition, the cilia disassembly activity of LPA was also observed in multiple other cell lines, including MEF and IMCD3 (Fig. 1g and Supplementary Fig. 1g). Thus, these results indicate that LPA is the major component in serum to trigger cilia disassembly.

### The LPA receptors (LPARs) are required for LPA-induced cilia disassembly

**Fig. 2:**
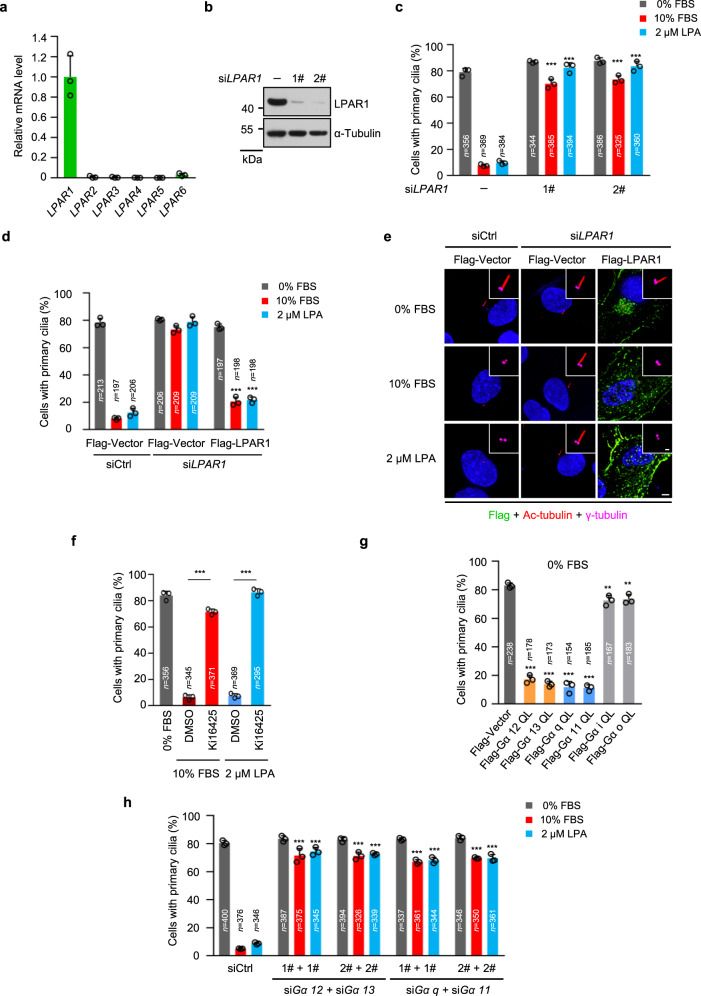
The LPA-LPAR pathway promotes cilia disassembly. **a** Expression of LPA receptors (LPARs) in RPE-1 cells. The mRNA levels of LPAR1-6 were determined by quantitative PCR (qPCR). **b**, **c** Knockdown of LPAR1 blocks the effect of serum- and LPA-induced cilia disassembly. RPE-1 cells were starved for 12 h and then transfected with control siRNA or *LPAR1* siRNAs respectively. Following 48 h serum starvation, cells were then treated with medium contained 10% FBS or 2 μM LPA for 24 h. **b** Immunoblotting shows the protein level of LPAR1 in LPAR1-knockdown RPE-1 cells. α-tubulin was used as a loading control. **c** Quantification of ciliation in RPE-1 cells. **d**, **e** Expression of Flag-LPAR1 resistant plasmid rescues cilia disassembly defects in LPAR1-depleted cells. **d** Quantification of ciliation in RPE-1 cells. **e** Representative images of RPE-1 cells in **d**. Cells were stained with anti-Flag (green), anti- Ac-tubulin (red) and anti-γ-tubulin (magenta) antibodies. Scale bar: 5 μm (main image) and 1 μm (magnified region). **f** LPAR1/3 antagonist Ki16425 blocks serum- and LPA-induced cilia disassembly. Ciliated RPE-1 cells were pretreated with Ki16425 (40 μM) or DMSO control for 30 min, and then cells were stimulated with 10% FBS or 2 μM LPA for 24 h. **g** The effect of Gα protein overexpression on cilia disassembly. RPE-1 cells were starved for 48 h, and then transfected with Flag-Gα plasmids expressing constitutively active Gα protein mutants (QL). **h** Knockdown of Gα 12/13 or Gα q/11 blocks the effect of serum- and LPA-induced cilia disassembly. RPE-1 cells were starved for 12 h and then transfected with control siRNA, a pool of siRNAs for *Gα 12* and *Gα 13*, or a pool of siRNAs for *Gα q* and *Gα 11*, respectively. Following 48 h serum starvation, cells were treated with 10% FBS or 2 μM LPA for 24 h. Source data are provided as a Source Data file. Three experiments were repeated independently with similar results in **b** and **e**. Data are presented as mean ± S.D. of three independent experiments in **a**, **c**, **d** and **f**–**h**. *n*, number of cells. **P < 0.01, ***P < 0.001. One-way ANOVA test was performed followed by Dunnett’s multiple comparisons in **g** or followed by Bonferroni’s multiple comparisons in **f**; two-way ANOVA test was performed followed by Dunnett’s multiple comparisons in **c**, **d** and **h**.

LPA acts through a family of high-affinity G-protein-coupled receptors, LPAR1-6^41^, only LPAR1 was highly expressed in RPE-1 cells (Fig. 2a). Knockdown of LPAR1 blocked the fetal bovine serum (FBS)- or LPA-induced cilia disassembly in RPE-1 cells (Fig. 2b, c). Serum or LPA treatment failed to induce the LPAR1-depleted cells to re-enter the cell cycle (Supplementary Fig. 2a). Importantly, the effect of FBS or LPA on cilia disassembly in ciliated, LPAR1-depleted RPE-1 cells was rescued by the expression of an small interfering RNA (siRNA)-resistant LPAR1 transgene (Fig. 2d, e and Supplementary Fig. 2b). Treatment of ciliated RPE-1 cells with Ki16425, an LPAR1 and LPAR3 antagonist^42^, markedly reduced serum- or LPA-induced cilia disassembly (Fig. 2f). These data indicate that the LPA-LPAR signal pathway is essential for serum- or LPA-triggered cilia disassembly. Intriguingly, knockdown of LPAR1 or addition of Ki16425 in RPE cells directly resulted in cilia formation in cycling cells cultured with 10% FBS (Supplementary Fig. 2c–e). One possibility is that the abolishment of the LPA inhibitory function might directly trigger the early events of cilium biogenesis, as suggested by a recent study^43^. Another possibility is that the assembly and disassembly of cilia are dynamically balanced processes. The disruption of disassembly can directly release the assembly activity. Consistent with this latter notion, the depletion of canonical disassembly factors, including Nek2 and Kif24, can also induce ciliogenesis in serum-cultured cells^14^.

LPARs bind to multiple heterotrimeric G proteins, such as Gα 12/13, Gα q/11 and Gα i/o and activate various downstream signals that mediate cell migration and growth^37,41^. Overexpression of constitutively active mutants (QL) of Gα 12/13/q/11, but not Gα i/o, markedly induced cilia disassembly in RPE-1 cells, even when these cells were serum-starved (Fig. 2g and Supplementary Fig. 2f, g). Conversely, the double knockdowns of Gα 12/13 or Gα q/11 blocked cilia disassembly in RPE-1 cells (Fig. 2h and Supplementary Fig. 2h). These results suggest that the LPA-LPAR pathway promotes cilia disassembly mainly through Gα 12/13 and Gα q /11.

### LPA pathway initiates cilia disassembly through Aurora A

**Fig. 3:**
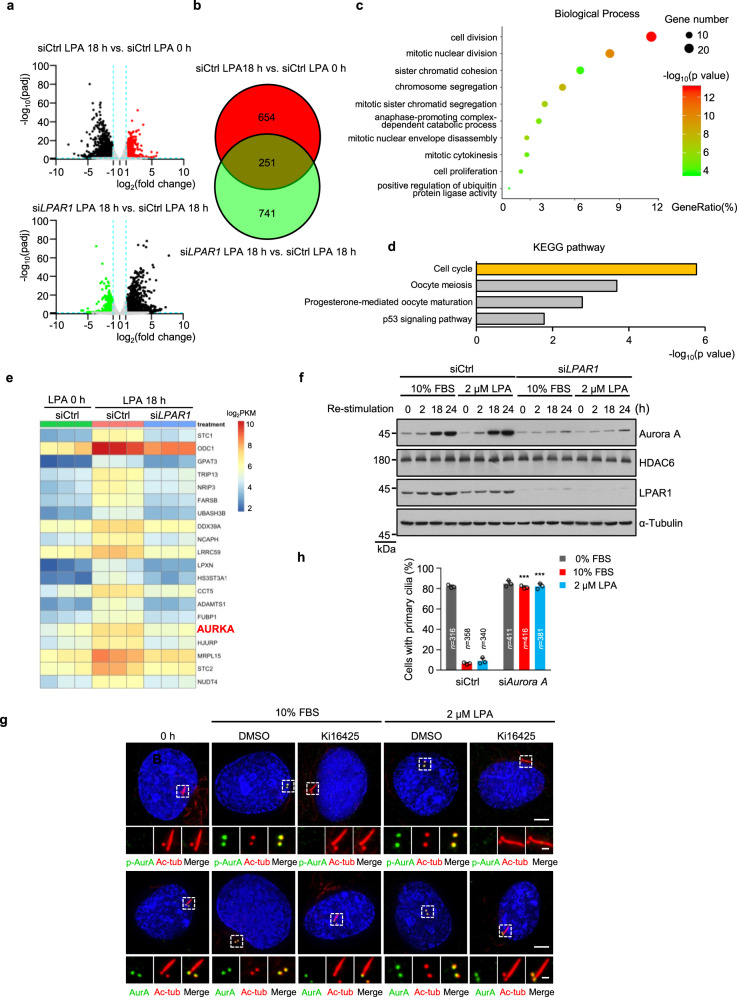
LPA signaling initiates cilia disassembly through Aurora A. **a** Scatter plot analysis of transcriptome expression profiles of siCtrl LPA 18 h versus siCtrl LPA 0 h, and si*LPAR1* LPA 18 h versus siCtrl LPA 18 h samples in RPE-1 cells. Red dots and green dots highlight the significantly upregulated or downregulated expressed genes, respectively. **b** The Venn diagram shows the overlap of upregulated and downregulated genes in **a**. **c** Statistics of enriched GO terms display 251 overlapped genes in (**b**, the brown part). The size of the point indicates the number of differentially expressed genes in this pathway, and the color of the points corresponds to a different p-value range. **d** Histogram of the enriched of KEGG pathway of 251 overlapped genes in (**b**, the brown part). **e** Heatmaps showing TOP20 enriched genes in (**b**, the brown part). **f** Immunoblot analysis was carried out using indicated antibodies. RPE-1 cells were starved for 12 h and then transfected with control siRNA or LPAR1 siRNA. Following serum starvation for another 48 h, cells were treated with 10% FBS or 2 μM LPA for indicated time points. **g** LPA activates Aurora A through phosphorylation in RPE-1. Ciliated RPE-1 cells were pretreated with Ki16425 (40 μM) or DMSO control for 30 min, and then cells were stimulated with 10% FBS or 2 μM LPA for 2 h. Cells were stained with anti-p-AurA (Aurora A, green) and anti-Ac-tub (Ac-tubulin, red) antibodies or anti-AurA (Aurora A, green) and anti-Ac-tub (Ac-tubulin, red) antibodies, respectively. Scale bar: 5 μm (main image) and 1 μm (magnified region). **h** The effect of serum- or LPA-induced cilia disassembly in Aurora A knockdown cells. Source data are provided as a Source Data file. Three experiments were repeated independently with similar results in **f** and **g**. Data are presented as mean ± S.D. of three independent experiments in **h**. *n*, number of cells. ***P < 0.001. Two way ANOVA test was performed followed by Dunnett’s multiple comparisons in **h**.

To decipher the mechanism underlying how LPA-LPAR pathway promotes cilia disassembly, we performed RNA-Seq to analyze the transcriptome changes in control and LPAR1-depleted cells with or without LPA treatment. By comparing gene expression levels in LPA-untreated or -treated samples, we found 2683 differentially expressed genes (905 upregulated and 1778 downregulated, |log_2_(fold change)| > 1, *p*_adj_ < 0.005) (Fig. 3a). We also compared gene expression levels in control or LPAR1-depleted cells after LPA treatment, and found 2532 differentially expressed genes (1540 upregulated and 992 downregulated, |log_2_(fold change)| > 1, *p*_adj_ < 0.005) (Fig. 3a). Based on these data, we next established a group of genes, which is upregulated in control cells after LPA treatment but is downregulated upon LPAR was depleted (Fig. 3b). Among these 251 genes, gene ontology (GO) classification of biological processes revealed a significant enrichment of several terms related to cell cycle (Fig. 3c), and KEGG signaling pathway analysis also revealed that the differentially expressed genes are predicted to be prominently involved in cell cycle pathways (Fig. 3d), which are consistent with our previous data (Supplementary Fig. 2a). Importantly, we found that *Aurora A*, a well-known gene involved in cilium disassembly, was significant upregulated by LPA in a LPAR-dependent manner (Fig. 3e). We then validated the mRNA expression of *Aurora A* and top two differentially expressed genes, *STC1* and *ODC1*, and found that these results were coincident with RNA-Seq (Supplementary Fig. 3a). We also detected that the Aurora A protein levels strongly accumulated at 18–24 h after serum or LPA stimulation^13^ (Fig. 3f), and this accumulation was greatly reduced in LPAR1-depleted cells (Fig. 3f).

A substantial body of evidence proved that the activation of Aurora A through phosphorylation occurred after serum stimulation, which plays a key role in cilia disassembly^13,44,45^. Next, we explored the effect of LPA on the activation of Aurora A. Consistent with the results of serum stimulation, phosphorylated Aurora A appeared at 2 h during ciliary disassembly following LPA treatment, and this phosphorylation can be blocked by LPAR1 inhibitor-Ki16425 (Fig. 3g). Given that the level of Aurora A has a little increase at 2 h after LPA treatment, we speculated that LPA might also be involved in the phosphorylation-mediated activation of Aurora A.

Since Aurora A plays an important role in cilia disassembly, we hypothesized that LPA may regulate cilia disassembly through Aurora A-HDAC6 signaling pathway. To test this hypothesis, we knocked down Aurora A in RPE-1 cells, and found that depletion of Aurora A abolished both serum- and LPA-induced cilia disassembly (Fig. 3h and Supplementary Fig. 3b). Histone deacetylase 6-HDAC6 as the downstream of Aurora A is well-known to deacetylate tubulin for depolymerizing the ciliary axoneme^13^. Next, we found the LPA-induced cilia disassembly was also markedly reduced after tubacin treatment (Supplementary Fig. 3c). Meanwhile, LPA treatment has no influence on the interaction between Aurora A and HDAC6 (Supplementary Fig. 3d). In addition, we also detected the influence on cilia disassembly of top two differentially expressed genes, *STC1* and *ODC1*. The data showed that these two genes have no significant effects on cilia disassembly (Supplementary Fig. 3e–h). Taken together, these data suggest that LPA induces cilia disassembly mainly through Aurora A signaling pathway.

### LPA signaling modulates Aurora A to trigger cilia disassembly through YAP/TAZ and calcium/CaM pathway

**Fig. 4:**
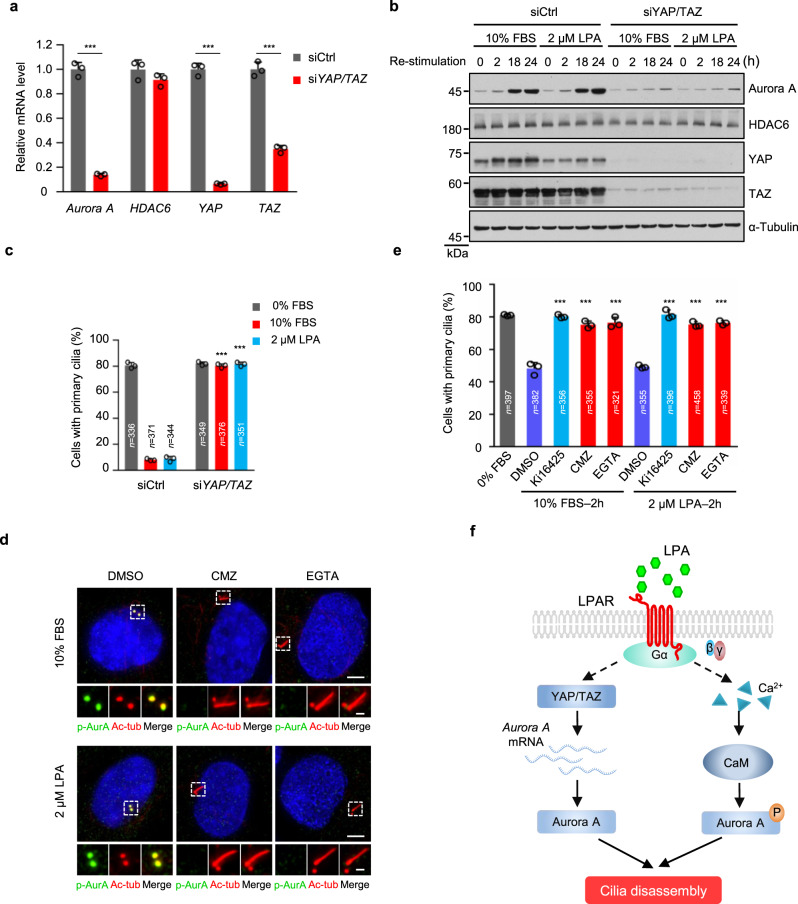
LPA signaling modulates Aurora A through YAP/TAZ and calcium/CaM pathway. **a** YAP/TAZ is required for the LPA-induced the transcription of *Aurora A*. RPE-1 cells were starved for 12 h and then transfected with control siRNA or *YAP/TAZ* siRNAs. Following serum starvation for another 48 h, cells were treated with 2 μM LPA for 18 h, and the mRNA levels were measured by qPCR. **b** Immunoblot analysis in control or YAP/TAZ knockdown cells using indicating antibodies. RPE-1 cells were transfected and treated as described in Fig. 3f. **c** The effect of serum- or LPA-induced cilia disassembly in control or YAP/TAZ knockdown cells. RPE-1 cells were transfected and treated as described in Fig. 2b, c. **d** CMZ or EGTA blocks serum- and LPA- induced Aurora A activation. Ciliated RPE-1 cells were pretreated with CMZ (5 μM), EGTA (0.5 mM) or DMSO control for 30 min, and then cells were stimulated with 10% FBS or 2 μM LPA for 2 h. Cells were stained with anti-p-AurA (Aurora A, green), anti-Ac-tub (Ac-tubulin, red) antibodies. Scale bar: 5 μm (main image) and 1 μm (magnified region). **e** CMZ or EGTA blocks serum- and LPA- induced cilia disassembly. Ciliated RPE-1 cells were pretreated with Ki16425 (40 μM), CMZ (5 μM), EGTA (0.5 mM) or DMSO control for 30 min, and then cells were stimulated with 10% FBS or 2 μM LPA for 2 h. **f** A proposed model for LPA signaling in the regulation of cilia disassembly. Source data are provided as a Source Data file. Three experiments were repeated independently with similar results in **b** and **d**. Data are presented as mean ± S.D. of three independent experiments in **a**, **c**, and **e**. *n*, number of cells. ***P < 0.001. Two-tailed Student’s *t*-test in **a**, two-way ANOVA test was performed followed by Dunnett’s multiple comparisons in **c** and **e**.

We next first investigated how LPA regulated the mRNA accumulation of Aurora A. LPA has been shown to inhibit Hippo pathway to activate YAP/TAZ^46^. A separate study has implicated YAP/TAZ in promoting the transcription of Aurora A^47^. We thus tested whether YAP/TAZ are involved in LPA-induced cilia disassembly. Strikingly, depletion of YAP/TAZ abolished the serum- or LPA-induced accumulation of Aurora A proteins and mRNAs (Fig. 4a, b). Consistent with previous studies^47^, both serum and LPA caused a significant nuclear accumulation of YAP in RPE-1 cells (Supplementary Fig. 4a). Furthermore, LPAR1 depletion reduced this nuclear accumulation (Supplementary Fig. 4a). More importantly, cilia disassembly triggered by LPA or serum was blocked by YAP/TAZ co-depletion (Fig. 4c and Supplementary Fig. 4b). These data suggest that LPA in serum acts through YAP/TAZ to elicit the transcription of Aurora A.

Next, we investigated how LPA regulate the phosphorylation of Aurora A. Since calcium/calmodulin (CaM) are required for Aurora A activation during cilia disassembly^44^ and LPA-LPA receptors can also induce Ca^2+^ mobilization in diverse cell types^48–50^, we thus detect whether Ca^2+^ signaling participate in the LPA-induced activation of Aurora A. As expected, we indeed observed the activation of Ca^2+^ signaling in ciliated RPE-1 cells after LPA treatment (Supplementary Fig. 4c). Importantly, the phosphorylation of Aurora A induced by LPA was blocked by the CaM inhibitor calmidazolium (CMZ), the calcium chelator EGTA (Fig. 4d). Importantly, the ability of LPA to induce cilia disassembly was also abolished by CaM inhibitor (Fig. 4e), suggesting that LPA-LPAR1-calcium/CaM was responsible for the phosphorylation of Aurora A and subsequent cilia disassembly. Taken together, these above data suggest that LPA activate Aurora A through calcium pathway and elicit the transcription of Aurora A through YAP/TAZ pathway, thereby initiating cilia disassembly (Fig. 4f).

### Lpar1 is required for the generation/maintenance of NPCs and proper neurogenesis

We next explored the physiological role of LPA-induced cilia disassembly in mice. *Lpar1* is enriched in mouse VZ/SVZ^51^. LPA and its major producing enzyme autotaxin (Atx) are present in cerebrospinal fluid (CSF) in rat^52^. We thus tested whether LPA-LPAR-induced cilia disassembly is involved in VZ/SVZ generation and expansion during mouse cortical development.

**Fig. 5:**
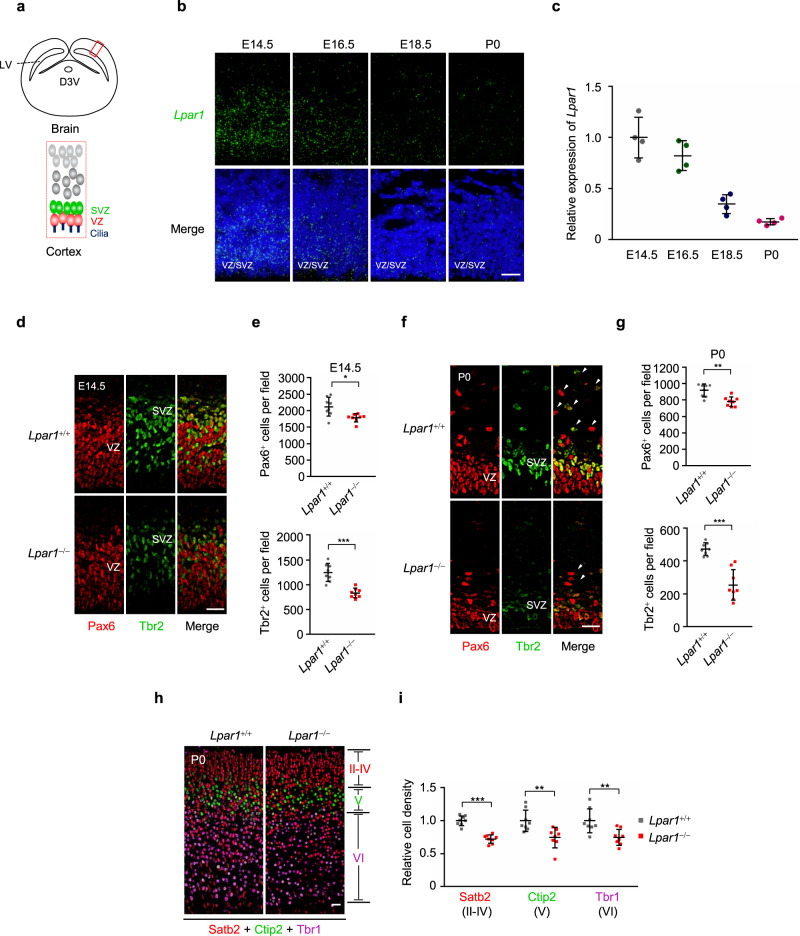
Lpar1 is required for proper neurogenesis during cortical development. **a** Schematic diagram of the VZ and SVZ in cerebral cortical. LV, lateral ventricles; D3V, dorsal 3rd ventricle; VZ, ventricular zone; SVZ, subventricular zone. **b** Expression of *Lpar1* in mouse cortex. RNAscope fluorescent in situ hybridization of *Lpar1* in cortex with wide-type mice at E14.5, E16.5, E18.5 and P0, *Lpar1* mRNA puncta (green), DNA (blue). Scale bar, 20 μm. **c** Quantification of *Lpar1* hybridization signals in **b**, *n* = 4 mice. **d**–**g** At E14.5 and P0, the total numbers of both Pax6^+^ and Tbr2^+^ cells are reduced in the *Lpar1*^*−*/−^ than in *Lpar1*^+/+^ mice cortex. **d**, **f** Representative images of *Lpar1*^+/+^ and *Lpar1*^*−*/−^ mice cortices at E14.5 (**d**) and P0 (**f**), which were stained with antibodies against Pax6 (red, radial glia cells marker) and Tbr2 (green, intermediate progenitor cells marker). White arrows indicate the neural progenitor cells (NPCs) expanded to the upper layers of cortex (**f**). Scale bar, 20 μm. **e**, **g** Quantification of Pax6^+^ cells and Tbr2^+^ cells at E14.5 (**e**) and P0 (**g**) cortices respectively, *n* = 8 sections from four mice. **h** *Lpar1*^*−*/−^ mice exhibited a reduction of cortical neuron density at P0. *Lpar1*^+/+^ and *Lpar1*^*−*/−^ cortices in mice were stained with layer II-IV marker (Satb2, red), layer V marker (Ctip2, green) and layer VI marker (Tbr1, purple). Scale bar, 20 μm. **i** Quantification of the relative cell density in **h**, *n* = 8 sections from four mice. Source data are provided as a Source Data file. Four experiments were repeated independently with similar results in **b**, **d**, **f** and **h**. Data are presented as mean ± S.D. in **c**, **e**, **g**, and **i**. *P < 0.05, **P < 0.01, ***P < 0.001. Two-tailed Student’s *t*-test.

We first verified the expression of *Lpar1* and *Atx* in mouse VZ/SVZ by RNAscope assay. We found that *Lpar1* mRNA was also enriched in the cerebral cortical VZ/SVZ niche. Interestingly, *Lpar1* expression in VZ/SVZ is developmentally regulated, reaching maximum at El4.5 and declining to very low levels at P0 (Fig. 5a–c). Furthermore, we found that both *Lpar1* and *Atx* were highly expressed in the RG cells of E14.5 mouse cerebral cortex (Supplementary Fig. 4a–c). Given that RG cells is close to the ventricle, we speculated that Atx protein might secrete into the CSF to synthesize LPA, by which Lpar1 is activated in the RG cells.

Previous studies showed that targeted deletion of Lpar1 results in ~50% neonatal lethality, impaired suckling in neonatal pups, and adult Lpar1 knockout survivors showing reduced size and craniofacial dysmorphism^32^. Importantly, both Lpar1 KO and Yap cKO mice showed obvious neurogenesis defects during cerebral cortex development, which was closely related to ciliogenesis^53–56^. We next examined the distribution patterns of NPCs in the developing cortex of *Lpar1-*knockout mice^32^. We detected radial glia (RG) cells in the VZ with Pax6 and intermediate progenitor cells (IPs) in the SVZ with Tbr2. Compared with the *Lpar1*^+/+^ control at E14.5 and P0, the total numbers of both Pax6- and Tbr2-positive cells were markedly reduced in the *Lpar1*^−/−^ cortex (Fig. 5d–g). These NPCs also failed to expand to upper layers in the *Lpar1*^−/−^ cortex at P0 (Fig. 5f). The NPCs from VZ/SVZ produce projection neurons for all layers of cerebral cortex. Decrease in the number of NPCs resulted in a reduction of cortical neuron density in the *Lpar1*^−/−^ cortex at P0 (Fig. 5h, i). Taken together, Lpar1 is required for generation or maintenance of NPCs and proper neurogenesis.

### Lpar1 modulates ciliogenesis and neurogenesis during cortical development

**Fig. 6:**
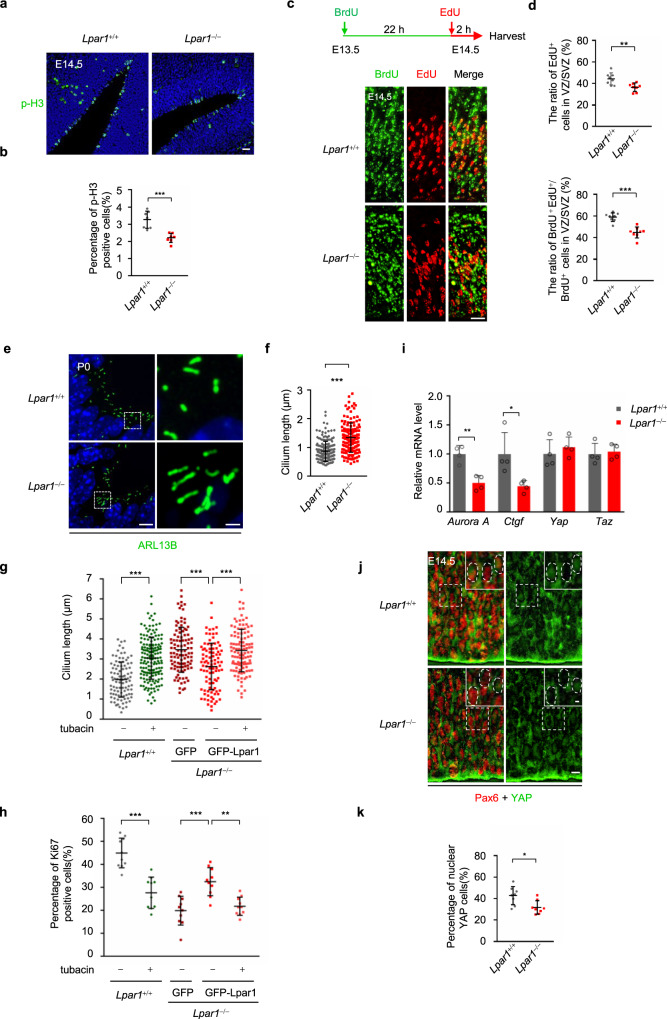
NPCs of *Lpar1*^*−*/−^ mice exhibit elongated cilia and decreased cell division. **a**, **b** VZ/SVZ cells in *Lpar1*^*−*/−^ mice exhibit a lower mitotic index. **a** Representative images of mitotic cells stained with phosphorylated histone 3 (p-H3, green) and DNA (blue) in E14.5 *Lpar1*^+/+^ and *Lpar1*^*−*/−^ mice cortices. Scale bar, 20 μm. **b** The percentage of p-H3-positive cells from VZ/SVZ cells in **a**, *n* = 8 sections from four mice. **c** Representative images of E14.5 *Lpar1*^+/+^ and *Lpar1*^*−*/−^ cortices subjected to dual pulse-chase labeling of BrdU (green) and EdU (red). The pulse-chase timing is shown on the top. Scale bar, 20 μm. **d** Quantification of the ratio of EdU^+^ cells in VZ/SVZ (top) and the ratio of BrdU^+^ EdU^+^ to BrdU^+^ cells in VZ/SVZ (down), *n* = 8 sections from four mice. **e** *Lpar1*^*−*/−^ RG cells possess longer cilia than *Lpar1*^+/+^. *Lpar1*^+/+^ and *Lpar1*^*−*/−^ cortices section at P0 were stained with cilia marker (ARL13B, green) and DNA (blue). Scale bar, 5 μm (main image) and 1 μm (magnified region). **f** Quantification of the cilium length in **e**, *Lpar1*^+/+^ː *n* = 179 cilia from four mice, *Lpar1*^*−*/−^ː *n* = 149 cilia from four mice. **g**, **h** Quantification of cilium length (**g**) or percentage of Ki67^+^ cells (**h**) in Sox2-positive RG cells. Isolated cells from *Lpar1*^+/+^ cerebral cortex were treated with 2 μM tubacin or DMSO for 72 h, and isolated cells from *Lpar1*^*−*/−^ cerebral cortex were infected with lentivirus expressed GFP or GFP-Lpar1 with or without 2 μM tubacin treatment for 72 h. **g** From left to right, *n* = 95, 164, 103, 111 cilia from four experiments. **h** *n* = 10 mice from four experiments. **i** qPCR analyzed the transcriptional levels of *Aurora A*, *Ctgf*, *Yap* and *Taz* in E14.5 *Lpar1*^+/+^ and *Lpar1*^*−*/−^ mice cortices. **j** Representative images of E14.5 *Lpar1*^+/+^ and *Lpar1*^*−*/−^ cortices stained with YAP (green) and PAX6 (red). Scale bars, 20 μm (main image) and 5 μm (magnified region). **k** Quantification of the percentage of PAX6^+^ cells with nuclear YAP in VZ. Source data are provided as a Source Data file. Four experiments were repeated independently with similar results in **a**, **c**, **e**, and **j**. Data are presented as mean ± S.D. in **b**, **d**, **f**, **g**, **h**, **i**, and **k**. *P < 0.05, **P < 0.01, ***P < 0.001. Two-tailed Student’s *t*-test in **b**, **d**, **f**, **i**, and **k**; One-way ANOVA test was performed followed by Bonferroni’s multiple comparisons in **g** and **h**.

Next, we evaluated whether *Lpar1* depletion influences the proliferation and self-renewal of NPCs. VZ/SVZ cells in *Lpar1*^−/−^ mice exhibited a lower mitotic index, suggesting that Lpar1 is required for the proliferation of NPCs (Fig. 6a, b). In addition, the apoptosis index was slightly increased in the *Lpar1*^−/−^ cerebral cortex as compared to *Lpar1*^+/+^ controls, which is consistent with previous study^53^ (Supplementary Fig. 5d, e). To test if *Lpar1*^−/−^ mice exhibited decreased self-renewal of NPCs, we performed a pulse-chase assay by sequential BrdU and EdU labeling in *Lpar1*^+/+^ and *Lpar1*^−/−^ mice (Fig. 6c). A smaller percentage of NPCs of *Lpar1*^−/−^ VZ/SVZ exhibited EdU incorporation, confirming the requirement of Lpar1 in NPC proliferation (Fig. 6c, d). The proportions of self-renewing NPCs (BrdU^+^EdU^+^ to BrdU^+^ cells) were significantly decreased in *Lpar1*^−/−^ mice as compared to *Lpar1*^+/+^ controls (Fig. 6c, d), indicating that Lpar1 is also required for NPCs self-renewal. Consistent with a requirement of LPAR1 for cilia disassembly, the length of cilia was significantly longer in *Lpar1*^−/−^ RG cells than that in *Lpar1*^+/+^ cells (Fig. 6e, f and Supplementary Fig. 5f). Moreover, knockdown of the negative regulator of cilia assembly (CP110, Cep97) in RPE-1 cells did not affect the cilium length^57^, but the cilium length was elongated when canonical disassembly factors were depleted^14^, which supports our notion that Lpar1 regulates ciliogenesis potentially through cilia disassembly mechanism.

To further confirm this notion, we isolated NPCs from *Lpar1*^+/+^ and *Lpar1*^−/−^ cerebral cortex and performed following experiments with or without overexpressing GFP-Lpar1. We found that overexpression of GFP-Lpar1 robustly rescued the elongated cilia and the reduction of NPCs proliferation in *Lpar1*^−/−^ NPCs (Fig. 6g, h and Supplementary Fig. 5g, h). In contrast, the rescued effect of GFP-Lpar1 was destroyed in *Lpar1*^−/−^ NPCs treated with tubacin (a HDAC6 inhibitor) (Fig. 6g, h and Supplementary Fig. 5g, h). Thus, these data suggest that LPA-Lpar1 pathway is essential for the proliferation of NPCs potentially by regulating cilia disassembly.

The transcription levels of the canonical cilia disassembly regulators, Aurora A were dramatically downregulated in RG cells in *Lpar1*^−/−^ mice (Fig. 6i). The mRNA expression of CTGF (a known LPA-inducible and YAP-dependent gene) was also obviously repressed in *Lpar1*^−/−^ mice (Fig. 6i). Moreover, we found that the nuclear localization of Yap was also significantly decreased in *Lpar1*^*−*/−^ mice compared to *Lpar1*^+/+^ controls (Fig. 6j, k), suggesting that the activity of Yap is decreased in *Lpar1*^*−*/−^ mice. Thus, these data indicate that LPA-LPAR-YAP pathway is required for the transcription of major cilia disassembly genes in mice.

Taken together, these results indicated that LPA-LPAR pathway is essential for the proliferation of NPCs in VZ/SVZ and subsequent neurogenesis potentially by regulating cilia dynamic and cell cycle progression during mouse embryo development. Further, recent studies reported that the knockout of ATX in the early zebrafish embryo results in defects in left right (L-R) patterning and the Kupffer’s vesicle (KV), which further support our findings^58,59^.

## Discussion

Cell-intrinsic regulatory mechanisms of cilia disassembly has been well-studied^12,60^. In contrast, extracellular signals that control cilia disassembly have remained elusive. Using serum as an entry point, our study has identified lysophosphatidic acid (LPA) as a physiological extracellular ligand that promotes cilia disassembly. The LPA pathway acts upstream of canonical disassembly modulators through activating a YAP/TAZ-dependent transcriptional program. Given that many ligands, such as estrogen, secretin and platelet-activating factor, can active YAP/TAZ acting through their receptor GPCRs^46^, it is likely that these GPCRs could similarly modulate ciliogenesis as LPA receptors. Future studies are needed to address these important issues under specific physiological and pathological contexts.

Activation of Aurora A kinase have been well-studied in cilia disassembly^13,16,44,61,62^, whereas the levels are also dramatically upregulated during cilia disassembly. Thus far, the mechanisms governing the dynamic regulation of Aurora A levels during the cilia life cycle remain unclear. Our study provides the explanation that the accumulation of Aurora A results from the increase of its transcription by LPA pathway-mediated activation of YAP/TAZ.

By systematic separation of components, we discovered LPA as the major factor in serum for driving cilia disassembly through Aurora A, while the recent work of Walia et al. identified a negative role for LPA in cilia assembly through Rab8/Rab11^43^. Actually, it is generally accepted that the assembly and disassembly of cilia are dynamically balanced processes. Previous study showed that depletion of canonical disassembly factors, including Nek2 and Kif24, could directly induce ciliogenesis in serum-cultured cells^14^. We also observed that knockdown of LPAR1 or Aurora A directly resulted in cilia formation in serum-cultured cells. Therefore, the disruption of disassembly can directly release the assembly activity and trigger the early events of cilium biogenesis even in the presence of serum, just as observed by Walia. Our findings together with Walia’s work will contribute to the comprehensive understanding about the extracellular signal controlling ciliogenesis, and LPA is expected to play important roles in many specific physiological and pathological contexts through regulating proper ciliogenesis.

NPCs in VZ/SVZ niche undergo both asymmetric and symmetric cell divisions for self-renewal and differentiation^29^. These divisions are regulated by developmental cues and extracellular signals. The primary cilia of these NPCs can detect diverse signaling factors in cerebrospinal fluid (CSF), such as sonic hedgehog (shh) and IGF^63,64^. On the other hand, the mitotic entry of NPCs requires the disassembly of their primary cilia^26–28^. Inactivation of cell-intrinsic cilia disassembly factors has been reported to cause abnormally elongated cilia and limit the proliferation of NPCs in VZ^65^. However, the extracellular factors that control cilia disassembly during cortical development are unknown. We show that LPA not only promotes cilia disassembly in cultured cells, but also regulates proper ciliogenesis during mammalian development. Our results now establish LPA as one such physiological extracellular factor in this process.

We propose that LPA in the CSF inhibits ciliogenesis through promoting cilia disassembly. However, the mouse embryo is so small that it is technically unfeasible to directly detect the presence of LPA in CSF during embryo development. We found that the *Atx* mRNA was highly expressed in the RG cells of E14.5 mouse VZ region, which is close to the ventricle (Supplementary Fig. 5a). Thus, we propose that as an exoenzyme, Atx protein is likely secreted into CSF to synthesize LPA in CSF, and this issue will be further analyzed in future studies.

Loss of LPA signaling through genetic ablation of LPAR1 causes elongated cilia and defective NPC proliferation and self-renewal. Interestingly, LPAR1 expression is enriched in NPCs and is developmentally regulated. Its expression level positively correlates with NPC proliferation. Its expression is high at E14.5 when NPCs undergo fast proliferation. Its expression is very low at P0 when NPC proliferation slows down. Therefore, we have discovered a developmentally regulated extracellular signaling that coordinates ciliogenesis and neurogenesis. All of the above results provide a different view into cilia disassembly during embryonic development and ciliopathy.

LPA production and LPARs expression are both elevated in many types of human cancers^66–70^. Elevated LPA and activation of LPARs have been shown to promote tumorigenesis in mouse models^38^. It is possible that defective ciliogenesis as a result of hyperactive LPA signaling is a causal factor of tumorigenesis. Targeting the LPA pathway with chemical inhibitors might be a potential strategy to counteract the ciliary defect caused by LPA and restrict the proliferation of cancer cells.

## Methods

### Mice

The generation and genotyping of *Lpar1*-deficient mice was described previously^32^. All animal procedures were approved by the Institutional Animal Care and Use. Animals were kept under specific-pathogen-free conditions, with controlled temperature (20–25 °C), humidity (40–60%) and light cycle (12 h light/dark). All embryos used for this study were obtained from natural mattings of virgin females 8-12 weeks old. Noon on the day of the discovery of a vaginal plug was considered to be embryonic day E0.5. P0 embryos were collected before the death of *Lpar1*^*−*/−^ pubs. All animal experiments were performed with the approval of the Institutional Animal Care and Use Committee of Military Medical Sciences (IACUC-DWZX-2019-506).

### Cell culture

All cell lines were maintained at 37 ^°^C with 5% CO_2_. hTERT RPE-1 (RPE-1) and IMCD-3 cells were kindly provided by Dr. Xueliang Zhu. HEK293T cells were obtained from the ATCC (CRL-3216). RPE-1 cells were cultured in DMEM/F-12 (1:1) supplemented with 10% FBS, 0.01 mg/mL hygromycin B and 1% penicillin/streptomycin, and IMCD-3 cells were cultured in DMEM/F-12 (1:1) supplemented with 10% FBS and 1% penicillin/streptomycin. HEK293T cells were cultured in DMEM supplemented with 10% FBS and 1% penicillin/streptomycin. Primary MEF cells were isolated from E14 wild C57BL/6J mouse embryos using standard protocol and incubated in DMEM medium supplemented with 15% FBS and 1% penicillin/streptomycin. For cilia formation, cells were incubated in DMEM/F12 (1:1) for 48 h; for cilia disassembly, ciliated cells were re-stimulated with DMEM/F12 (1:1) contained with serum, LPA or other reagents as indicated.

### Immunofluorescence staining and imaging

To detect primary cilia, Aurora A and p-Aurora A, cells were placed on ice for 10 min, and then fixed and permeabilized in cold methanol for 5 min. Cells transfected with plasmids were fixed in cold methanol for 30 s. To visualize the localization of YAP and detect primary cilia and Ki67 in isolated NPCs, cells were fixed with 4% paraformaldehyde (PFA) for 10 min and permeabilized with 0.1% Triton X-100 in PBS.

For tissue immunofluorescence, brains were fixed in 4% PFA at 4 °C overnight and then embedded in paraffin (Thermo Fisher Scientific) or OCT compound (Sakura). Paraffin-embedded sections were serially cut at 3 μm. Frozen sections were cut at 8 μm.

Cells and tissue sections were blocked with 3% BSA in PBS for 1 h prior to incubation with primary antibodies overnight at 4 °C. Secondary antibodies used were Alexa Fluor 488-, 546- and 647- conjugated goat anti-mouse, anti-rabbit, anti-rat or donkey anti-goat IgG (Thermo Fisher Scientific). To detect the overexpression of exogenous plasmid, cells were stained with ANTI-FLAG^®^ M2-FITC antibody for 1 h at room temperature. DNA was stained with Hoechst 33342 (1:500, H3570, Thermo Fisher Scientific) for 10 min at room temperature. Slides were then washed three times and mounted. EdU staining was performed using the Cell-Light EdU Apollo567 In Vitro Kit in accordance with the manufacturer’s instruction (C10310-1, RIBOBIO). Ca^2+^ signaling was detected using the Fluo-4, AM in accordance with the manufacturer’s instruction (F8500, Solarbio).

Immunofluorescence was detected using 60×/1.42 Oil objective (Figs. 1–4, Supplementary Figs. 1–4) or 20×/0.75 objective (Figs. 5, and 6; and Supplementary Fig. 5) with a DeltaVision (DV) Image Restoration Microscope, a 63×/1.40 oil objective on Zeiss LSM 880 (Fig 6, and Supplementary Fig. 5) or a 63×/1.40 Oil objective on Zeiss LSM 880 with airyscan (Fig. 6 and Supplementary Fig. 5). For data acquisition on a DV Image Restoration Microscope, we used GE healthcare SoftWoRx 6.5.2. For data collection on Zeiss LSM 880 microscope, we used ZEN 2.1 SP2 Black version 13.0.2.518 (ZEISS). All acquisition settings were kept constant for experimental and control groups in the same experiment. The representative images acquired by DV system were processed by iterative constrained deconvolution (SoftWoRx, Applied Precision Instruments). All raw images were analyzed with Volocity 6.0 software (Perkin Elmer). Cilium length was measured from the tip to the base.

### Antibodies

Antibodies used in this study included mouse anti-Ac-tubulin antibody (1:800, T6793, Sigma), rabbit anti-γ-Tubulin antibody (1:600, T6557, Sigma), mouse anti-LPAR1 antibody (1:100, sc-515665, Santa), mouse anti-α-Tubulin (1:5000, T5168, Sigma), mouse anti-Flag antibody (1:1000, F3165, Sigma), mouse anti-Gα 12 antibody (1:200, sc-515445, Santa), mouse anti-Gα 13 antibody (1:200, sc-293424, Santa), mouse anti-Gα q antibody (1:200, sc-136181, Santa), mouse anti-Gα 11 antibody (1:200, sc-390382, Santa), mouse anti-YAP antibody (1:1000, sc-101199, Santa), rabbit anti-TAZ antibody (1:4000, 66500-1-Ig, Proteintech), rabbit anti-Aurora A antibody (1:1000, 4718s, Cell Signaling Technologies), rabbit anti-HDAC6 antibody (1:1000, 7558T, Cell Signaling Technologies), rabbit anti-ARL13B antibody (1:400, 17711-1-AP, Proteintech), mouse anti-BrdU antibody (1:250, 11-286-C100, Exbio), rabbit anti-Pax6 antibody (1:500, PRB-278P, Covance), rat anti-Tbr2 antibody (1:500, 14-4875-82, Thermo Fisher Scientific), rabbit anti-Satb2 antibody (1:500, ab92446, Abcam), mouse anti-Tbr1 antibody (1:250, 66564-1-Ig, Proteintech), rabbit anti-Ctip2 antibody (1:250, ab28448, Abcam), rabbit anti-p-H3(Ser10) antibody (1:500, 9701s, Cell Signaling Technologies), rabbit anti-Phospho-Aurora A (Thr288) antibody (1:100, MA5-14904, Thermo Fisher Scientific), goat anti-Sox2 antibody (1:300, Sc-17320, Santa), rabbit anti-Ki67 antibody (1:500, 9129T, Cell Signaling Technologies), rabbit anti-cleaved Caspase3 antibody (1:400, 9664, Cell Signaling Technologies), mouse anti-Flag-M2 FITC (1:8000, F4049, Sigma), Goat anti-Mouse Alexa Fluor 488 (1:500, A11029, Thermo Fisher Scientific), Goat anti-Mouse Alexa Fluor 546 (1:500, A11030, Thermo Fisher Scientific), Goat anti-Mouse Alexa Fluor 647 (1:500, A21235, Thermo Fisher Scientific, lot: 2088736), Goat anti-Rabbit Alexa Fluor 488 (1:500, A11034, Thermo Fisher Scientific), Goat anti-Rabbit Alexa Fluor 546 (1:500, A11035, Thermo Fisher Scientific), Goat anti-Rabbit Alexa Fluor 647 (1:500, A21245, Thermo Fisher Scientific), Goat anti-Rat Alexa Fluor 555 (1:500, A21434, Thermo Fisher Scientific), Goat anti-Rat Alexa Fluor 488 (1:500, A11006, Thermo Fisher Scientific), Donkey anti-Goat Alexa Fluor 647 (1:500, A21447, Thermo Fisher Scientific).

### Chemicals

LPA (18:1, L7260), phosphatidylserine (PS, P7769-5MG) and phosphatidylcholine (PC, P3556-25MG) were purchased from Sigma and all other lipid chemicals were purchased from Avanti Polar Lipids. Other Lipids shown in Fig. 1D and Fig. S1D are lyso phosphatidylserine (LPS, 860081), lyso-phosphatidylcholine (LPC, 850092P), phosphatidylethanolamine (PE, 840465P), lyso phosphatidylethanolamine (LPE, 858127c), phosphatidylglycerol (PG), lyso phosphatidylglycerol (LPG, 830071), phosphatidic acid (PA, 840861P), sphingomyelin (SPH, 860062P), phosphatidylinositol (PI, 850142P), phosphatidylinositol 3-phosphate (PI_3_P, 850150P), phosphatidylinositol(3,4)-bisphosphate (PI_3,4_P, 850153P), cardiolipin (CL, 710335P), 16:0 LPA (857123P), 18:0 LPA (857125c) and 20:4 LPA (857128p).

The LPAR1/3 inhibitor Ki16425 (40 μM, S1315, Selleck), HDAC6 inhibitor tubacin (2 μM, HY-13428, MCE), CaM inhibitor CMZ (5 μM, 2561, R&D), calcium chelator EGTA (0.5 mM, HY-D0973, MCE) or DMSO vehicle were added to RPE-1 cells 30 min prior to the treatment of serum or LPA.

### RNA extraction, reverse transcription, and real-time PCR

Following various treatments, cells or mouse cerebral cortex were washed with cold phosphate-buffered saline and subjected to RNA extraction using TRIZOL (93289, Sigma). RNA samples were reverse-transcribed to complementary DNA (cDNA) using the PrimeScript™ RT Master Mix (RR036A, Takara), cDNA was then diluted and used for quantification (with GAPDH gene as a control) by real-time PCR, which was performed on an Roche StepOnePlus Real-Time PCR System and analyzed using the 2^−ΔΔCt^ method. Primers pairs used in this study are: *LPAR1* (Gene ID 1902), GTGTGGGCTGGAACTGTAT CTG/TAGTCCTCTGGCGAACATAG; *LPAR2* (Gene ID 9170), GGCCAGTGCTACTACAACGAGACC/TGGAGGCGATGGCTGCTATG AC; *LPAR3* (Gene ID 23566), CCTGGTGGTTCTGCTCCTCGAC/GTGCCATACAT GTCCTCGTCCTTG; *LPAR4* (Gene ID 2846), ATTGAAGTTGTTGGGTTTATCAT/G CACAAGGTGATTGGGTACAT; *LPAR5* (Gene ID 57121), CCTGGCGGCGGTGGT CTACTCGTC/GACCGCCAGCGTGCTGTTGTAGGG; *LPAR6* (Gene ID 10161), TTGTATGGGTGCATGTTCAGC/GCCAATTCCGTGTTGTGAAGT; *Aurora A* (Gene ID 6790), GAGGTCCAAAACGTGTTCTCG/ACAGGATGAGGTACACTGG TTG; *STC1*(Gene ID 6781), GGCGACCACCAAAGTCAAAC/TACTTGTCGCAT TGGGGTCC; *ODC1*(Gene ID 4953) GGGCGCTCTGAGATTGTCAC/GGCAATC CGCAAAACCAACT; *HDAC6* (Gene ID 10013), AAGAAGACCTAATCGTGGGAC T/GCTGTGAACCAACATCAGCTC; *YAP1* (Gene ID 10413), TAGCCCTGCGTAGC CAGTTA/TCATGCTTAGTCCACTGTCTGT; *TAZ* (Gene ID 25937), GATCCTGCC GGAGTCTTTCTT/CACGTCGTAGGACTGCTGG; *GAPDH* (Gene ID 2597), GGA GCGAGATCCCTCCAAAAT/GGCTGTTGTCATACTTCTCATGG; *Aurora A* (Gene ID 20878), CTGGATGCTGCAAACGGATAG/CGAAGGGAACAGTGGTCTTAAC A; *Ctgf* (Gene ID 14219), GGGCCTCTTCTGCGATTTC/ATCCAGGCAAGTGCAT TGGTA; *Yap1* (Gene ID 22601), TACTGATGCAGGTACTGCGG/TCAGGGATC TCAAAGGAGGAC; *Taz* (Gene ID 97064), GAAGGTGATGAATCAGCCTCTG/G TTCTGAGTCGGGTGGTTCTG; *Gapdh* (Gene ID 14433), AGGTCGGTGTGAACG GATTTG/TGTAGACCATGTAGTTGAGGTCA.

### Cloning and plasmids

The resistant plasmid expressing recombinant pCDNA3.0-Flag-LPAR1 was synthesized BioMed Company. The plasmids Gα 12 QL, Gα 13 QL, Gα q QL, Gα i QL, Gα o QL were obtained from Dr. Faxing Yu, and the cDNA of Gα 11 was obtained from Dr. Jiahuai Han. Flag- Gα 12 QL, Flag-Gα 13 QL, Flag-Gα q QL, Flag-Gα i QL, Flag-Gα o QL were amplified by PCR and cloned into pCDNA3.0, and pCDNA3.0-Flag-Gα 11 Q209L mutant were generated by PCR-based site-directed mutagenesis. All constructs were verified by DNA sequencing. Plasmid transfection into RPE-1 cells was performed using Lipofectamine™ LTX Reagent (Thermo Fisher Scientific) according to the manufacturer’s instruction. Lentivirus expressing GFP or GFP-Lpar1 was purchased from Taitool Bioscience.

### RNAi

Synthetic small interfering RNA oligonucleotides were synthesized by Sigma or Life Technologies. Transfection of siRNAs using RNAiMAX (Thermo Fisher Scientific) was performed according to the manufacturer’s instructions. The sequences of siRNAs are as follows. control siRNA: 5′-UUCUCCGAACGUGUCACGUTT-3′; *LPAR1* siRNA (1#, sigma): 5′-GAAAUGAGCGCCACCUUUAdTdT′; *LPAR1* siRNA (1#, Thermo Fisher Scientific): 5′-CAUCUGCUGGACUCCUGGAUUGGUU-3′; *Gα 12* siRNA (1#, sigma): 5′-GAGCAUGACUUCGUUAUUAdTdT-3′; *Gα 12* siRNA (2#, sigma): 5′-CGGUGAAGUACUUCCUGGAdTdT-3′; *Gα 13* siRNA (1#, sigma): 5′-CU AUGACCGGCGUCGAGAAdTdT-3′; *Gα 13* siRNA (2#, sigma): 5′-GUAUGAGGGU GCUGGUUGAdTdT-3′; *Gα q* siRNA (1#, sigma): 5′-CUCAAGAUCCCAUACAAGU dTdT-3′; *Gα q* siRNA (2#, sigma): 5′-CAAUAAGGCUCAUGCACAAdTdT-3′; *Gα 11* siRNA (1#, sigma): 5′-GAGUUCAUCCUGAAGAUGUdTdT-3′; *Gα 11* siRNA (2#, sigma): 5′-CCAAGCUCGUCUACCAGAAdTdT-3′; *STC1* siRNA(sigma): 5′-CCAAC AGAUACUAUAACAGdTdT-3′; *ODC1* siRNA(sigma): 5′-GAGATCACCGGCGTA ATCAdTdT-3′; *YAP1* siRNA (Thermo Fisher Scientific): 5′-CAGCAGAAUAU GAUGAACUCGGCUU-3′; *TAZ*(*WWTR1*) siRNA (Thermo Fisher Scientific): 5′-GAGAAAGGAUUCGAAUGCGCCAAGA-3′; *Aurora A* siRNA (Thermo Fisher Scientific): 5′- GGGUAAAGGAAAGUUUGGUAA UGUU-3′.

### Immunoprecipitation

For immunoprecipitation (IP) experiments, indicated plasmids were transfected into HEK293T cells. At 6 h post-transfection, cells were treated with 40 μM Ki16425 or DMSO for 24 h. Cells were then lysed in M2 buffer (50 mM Tris-HCl, pH 7.5, 1% NP-40, 150 mM NaCl, 0.5 mM EGTA, 0.5 mM EDTA, 1 mM DTT, 1.5 mM MgCl_2_, 1 mM PMSF), and incubated with anti-Flag M2 affinity gel (A2220, Sigma) for 4 h at 4 °C. The immunoprecipitated proteins were immunoblotted with indicated antibodies.

### Western blot

Cell lysates were prepared in M2 buffer (20 mM Tris-HCl, pH 7.5, 250 mM NaCl, 3 mM EDTA, 3 mM EGTA, 0.1% NP-40) with complete protease inhibitor cocktail (Roche). Protein isolated from cell lysates was quantified with the Bradford assay (Biorad). Protein was boiled at 95 °C for 5 min prior to being loaded into a 4–12% Bis-Tris Protein Gels. Proteins were transferred to PVDF membrane, blocked with 5% non-fat dry milk (NFDM) in Tris-buffered saline containing 0.1% Tween 20 (TBST) for 1 h at room temperature and probed overnight with primary antibodies in 5% bovine serum albumin in TBST at 4 °C (under constant shaking on a rocker). After washing three times in TBST for 5 min each, the membrane was incubated with secondary antibodies at 1:5,000 dilution in 5% NFDM/TBST for 1 h at room temperature. Membranes were washed three times in TBST, developed with Clarity Western ECL Substrate (Thermo Fisher Scientific). Images have been cropped for presentation (uncropped scans of the blots were shown in Source Data file).

### RNA-seq experiments and analysis

RPE-1 cells were starved for 12 h and then transfected with control siRNA or LPAR1 siRNAs, respectively. Following 48 h serum starvation, cells of siCtrl group were collected (siCtrl LPA 0 h), and siCtrl or siLPAR1 cells were both treated with medium contained 2 μM LPA for 18 h (siCtrl LPA 18 h and siLPAR1 LPA 18 h). Total RNAs with polyA tails of these three groups of RPE-1 cells were purified, reverse-transcribed to cDNA, and sequenced. Sequenced reads for each sample were aligned to hg38 with STAR 2.6.0a^71^ and differentially expressed genes (DEGs) were identified with DESeq2^72^. We compared samples between siCtrl LPA 18 h and siCtrl LPA 0 h groups or siLPAR1 LPA 18 h and siCtrl LPA 18 h groups, and DEGs were filtered with *p*_adj_ < 0.05 and |log2(fold change)| > 1. DAVID^73^ (v6.8, https://david.ncifcrf.gov/) was used to perform gene functional annotation clustering (Gene Ontology: GOTERM_BP_FAT; Pathway: KEGG_PATHWAY).

### Thymidine analogs injection

Solutions of 1 mg/mL 5-ethynyl-2′-deoxyuridine (EdU) (Thermo Fisher Scientific) and 10 mg/mL bromo-deoxyuridine (BrdU) (Thermo Fisher Scientific) were prepared in PBS solution, pH 7.2. BrdU (50 mg/kg) and EdU (5 mg/kg) were injected intraperitoneally into the dam mice at 24 h and 2 h, respectively, before euthanasia.

### RNAscope in situ hybridization

We used an RNAscope Multiplex Fluorescent Reagent Kit (320850, ACDbio) and *Lpar1* target probes (318501, ACDbio) or *Atx* target probes (402441-C3, ACDbio) for mouse in situ hybridization of *Lpar1* or *Atx* mRNA. The experiment was performed according to the manufacturer’s instructions.

### Isolation and culture of NPCs in cerebral cortex

The cerebral cortexes were dissected out from P0 *Lpar1*^+/+^ or *Lpar1*^−/−^ embryos. NPCs and other cells in the cortex were isolated in accordance with the manufacturer’s instructions (130-092-628, Miltenyi Biotec). Isolated cells were resuspended in Dulbecco’s modified Eagle medium (DMEM) containing glutamine, penicillin/streptomycin, sodium pyruvate, l-cysteine and B27, and plated onto coverslips coated with poly-lysine in 24-well plates^56^. The cultures were maintained at 37 °C with 5% CO_2_. Cells were then infected with lentivirus expressing GFP or GFP-Lpar1 (titer of 2 × 10^6^ TU/mL) with or without 2 μM tubacin treatment. About 72 h later, cells were fixed and analyzed for cilia length and cell cycle procession.

### Statistics and reproducibility

Statistical calculations were performed with SPSS 21.0 software. The Shapiro-Wilk test was used to test the normality and Levene’s test was applied to test the homogeneity of variance. For the data of two groups that meet the normal distribution, unpaired two-tailed *t*-test was applied for the comparisons with siCtrl, 0% FBS control or as indicated in figure legends. For the data of two groups that do not meet the normal distribution, we used Mann–Whitney test. Multiple comparisons were carried out by using one-way or two-way ANOVA followed by Bonferroni’s or Dunnett’s multiple comparisons, as noted in the figure legends. For all tests, differences were considered statistically significant if *P*-values were less than 0.05 (as indicated with *, *P < 0.05; **P < 0.01; ***P < 0.001). No statistical methods were used to predetermine sample size. The experiments were not randomized. No samples were excluded. The investigators were blinded to assess all the staining assays.

### Reporting summary

Further information on research design is available in the Nature Research Reporting Summary linked to this article.

## Supplementary information

Supplementary Information

Reporting Summary

## Data Availability

All RNA sequencing files were deposited in the short read sequence archive under BioProject ID PRJNA678549. Differentially expressed genes were also mapped to the KEGG database (https://www.kegg.jp/) for pathway analysis. The authors declare that all data supporting the findings of this study are available within the article and its Supplementary Information files, or from the corresponding authors upon reasonable request. Source data are provided with this paper.

## Source data

Source Data
