# Supplementary material for: LPA signaling acts as a cell-extrinsic mechanism to initiate cilia disassembly and promote neurogenesis
Source: Nat Commun. 2021 Jan 28;12:662. doi: 10.1038/s41467-021-20986-y (PMC7843646; doi:10.1038/s41467-021-20986-y)
Supplement: Supplementary file 1 — Supplementary Information [file 41467_2021_20986_MOESM1_ESM.pdf]

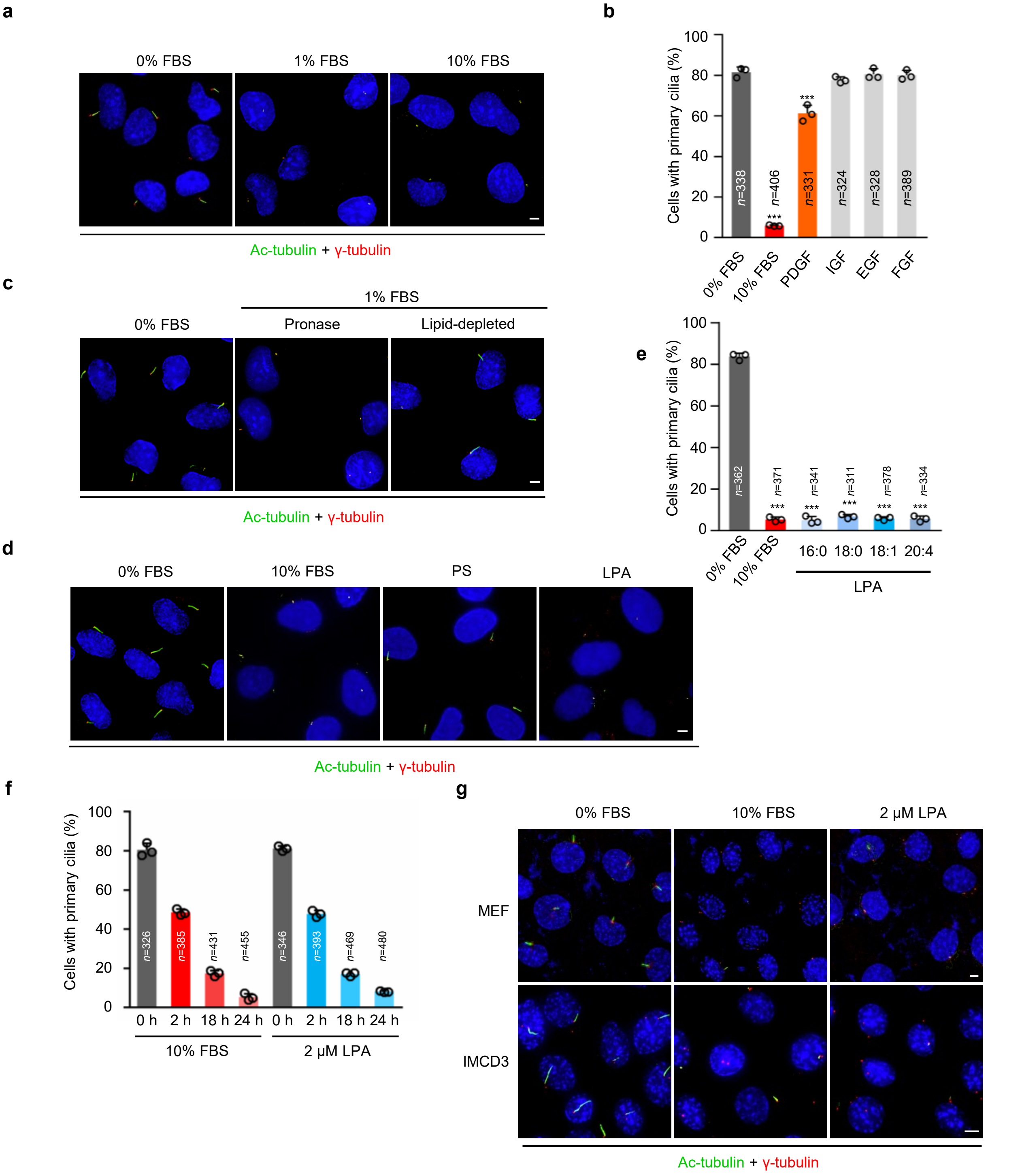

Supplementary Fig. 1 LPA induces cilia disassembly in human and mouse cell lines. **a**, **c**, **d** Representative images of RPE-1 cells in Fig. 1b-1d. Cells were stained with anti-Ac-tubulin (green) and anti-γ-tubulin (red) antibodies. Scale bar, 5 μm. **b** Effect of growth factors on cilia disassembly. RPE-1 cells were starved for 48 h, and then stimulated with 10% FBS, PDGF (100 ng/ml), IGF (10 μM), EGF (100 ng/ml) or FGF (100 ng/ml) for 24 h. **e** Effect of LPA isoforms on cilia disassembly. RPE-1 cells were starved for 48h, and then stimulated with 2 μM LPA isoforms with different lengths and degrees of saturation of the fatty acid tails as indicated for 24 h. **f** Time-dependent effect of LPA on cilia disassembly. Ciliated RPE-1 cells were treated with 10% FBS or 2 μM LPA as indicated time points. **g** Representative images of RPE-1 cells in Fig. 1g. Cells were stained with anti-Ac-tubulin (green) and anti-γ-tubulin (red) antibodies. Scale bar, 5 μm. Source data are provided as a Source Data file. Three experiments were repeated independently with similar results in (**a**), (**c**), (**d**) and (**g**). Data are presented as mean ± S.D. of three independent experiments in (**b**), (**e**) and (**f**). *n*, number of cells. ***$P < 0.001$. One way ANOVA test was performed followed by Dunnett's multiple comparisons in in (**b**) and (**e**).

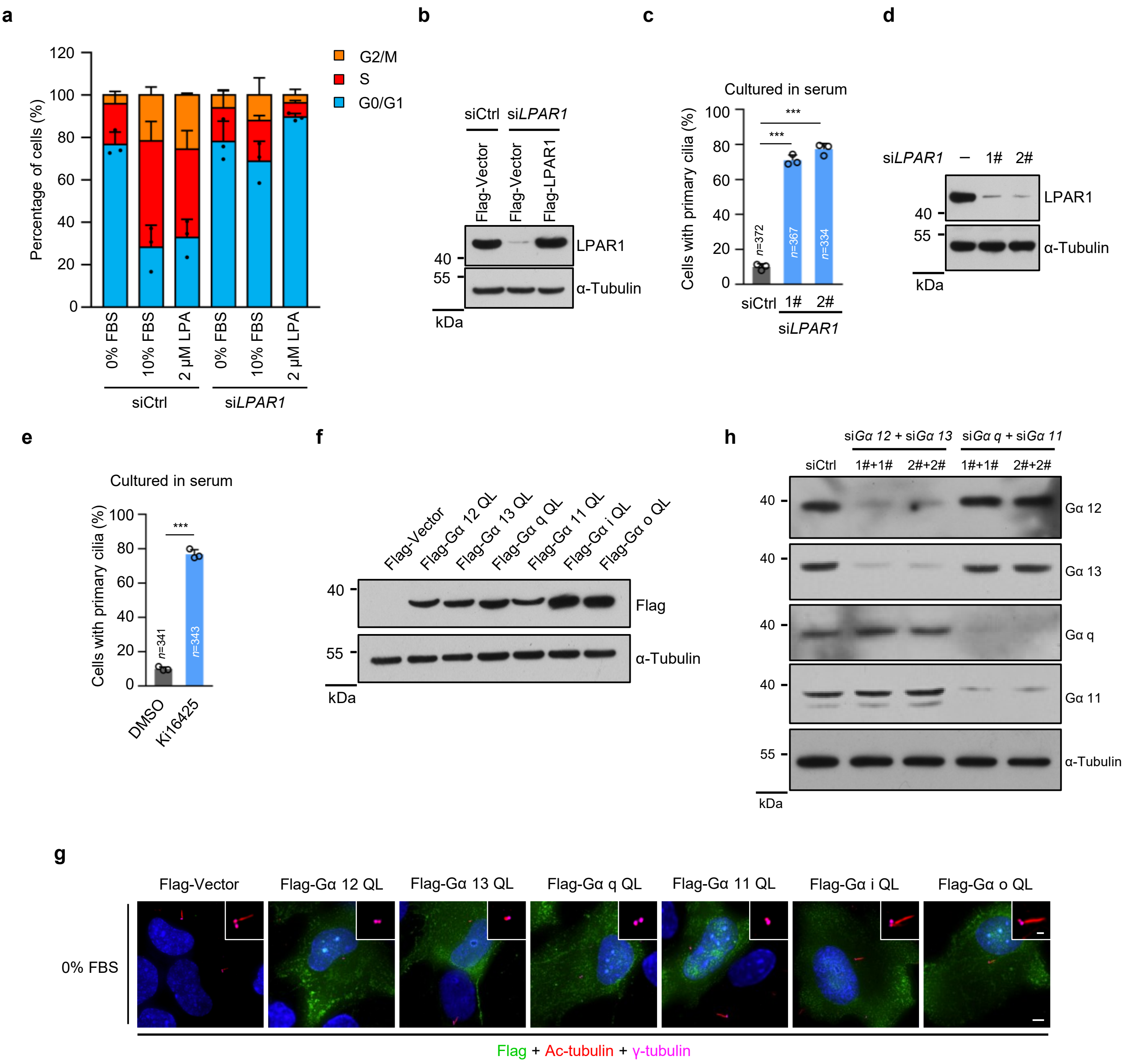

Supplementary Fig. 2 LPA signaling is required for cilia disassembly. **a** Cell cycle analysis was performed on RPE-1 cells, which were transfected and treated as described in Fig. 2C. FACS analysis of G0/G1, S, and G2/M cells by nuclear Propidium Iodide (PI) staining. **b** Immunoblots of RPE1-1 cell lysates in Fig. 2d with the indicated antibodies. **c**, **d** Knockdown of LPAR1 directly induces cilia formation. RPE-1 cells cultured in medium containing 10% FBS were transfected with control or *LPAR1* siRNA for 48 h, and then stained for cilia. (**c**) Quantification of ciliation in RPE-1 cells. (**d**) Immunoblotting shows the protein level of LPAR1 in LPAR1-knockdown RPE1 cells. α-tubulin was used as a loading control. **e** Inhibition of LPAR1 directly induces cilia formation. RPE-1 cells were treated with Ki16425 (40 μM) for 48 h in the presence of serum (10% FBS). **f**, **h** Immunoblots of RPE1-1 cell lysates in Fig. 2g and 2h with the indicated antibodies. **g** Representative images of RPE-1 cells in Fig. 2g. Cells were stained with anti-FITC-Flag (green), anti-Ac-tubulin (red) and anti-γ-tubulin (magenta) antibodies. Scale bar: 5 μm. Source data are provided as a Source Data file. Three experiments were repeated independently with similar results in (**b**), (**d**), (**f**), (**h**) and (**g**). Data are presented as mean ± S.D. of three independent experiments in (**a**), (**c**) and (**e**). *n*, number of cells. ****P* < 0.001. Two-tailed Student's *t*-test in (**e**); one way ANOVA test was performed followed by Dunnett's multiple comparisons in (**c**).

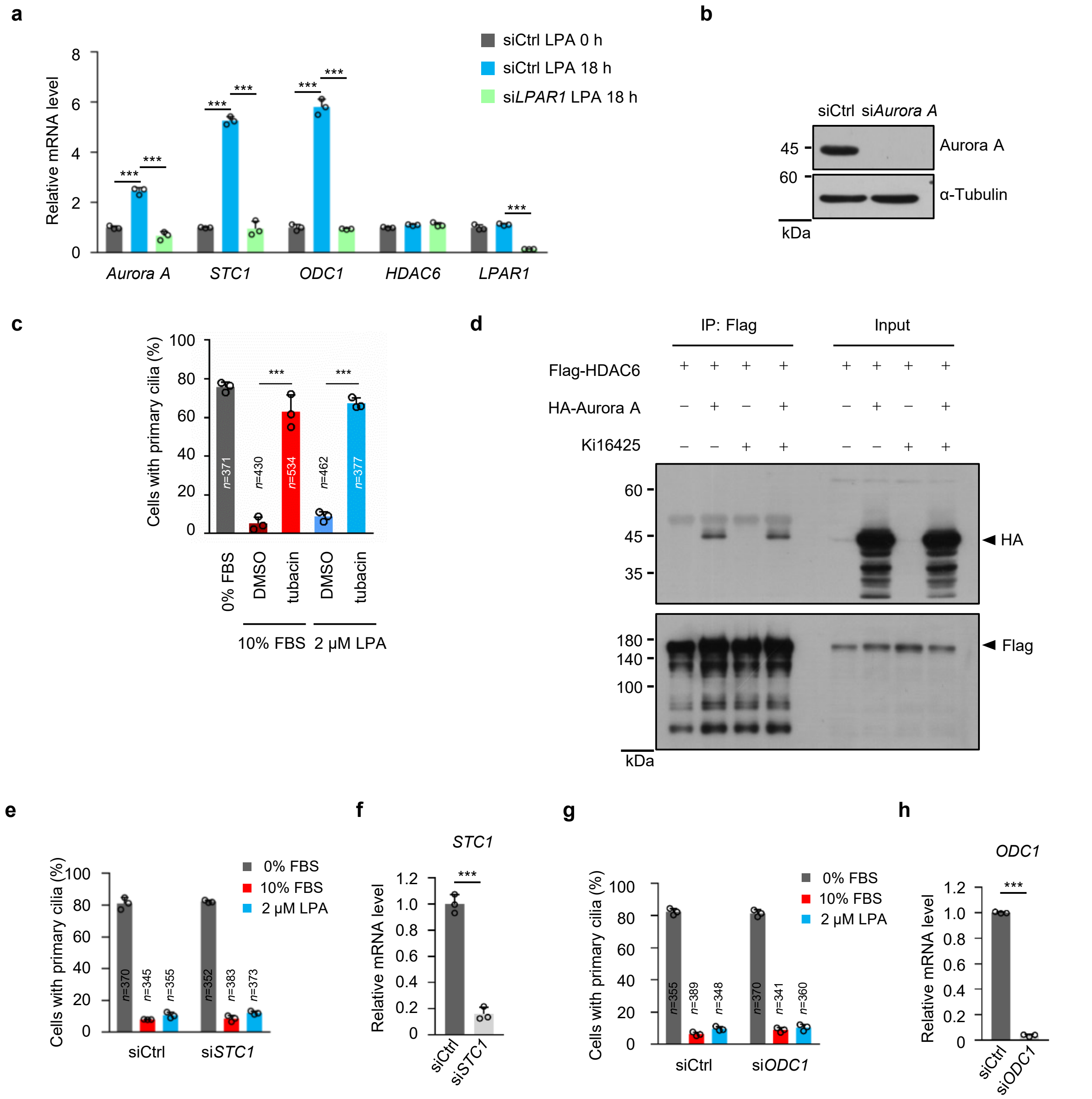

Supplementary Fig. 3 LPA induces cilia disassembly through Aurora A-HDAC6 pathway. **a** Quantitative real-time PCR-analyzed transcription of *Aurora A*, *STC1* and *ODC1*. Value was normalized to GAPDH gene expression. RPE-1 cells were transfected and treated as described in Fig. 2b and 2c. **b** Immunoblots of RPE-1 cell lysates in Fig. 3g with the indicated antibodies. **c** HDAC6 inhibitor tubacin blocks serum- and LPA-induced cilia disassembly. Ciliated RPE-1 cells were pretreated with tubacin (2 μM) or DMSO control for 2 hours, and then cells were stimulated with 10% FBS or 2 μM LPA for 24 h. **d** Immunoblot analysis of the interaction between Aurora A and HDAC6. HEK293T cells were transfected with indicated plasmids, and then treated with DMSO or Ki16425 (40 μM) as indicated. **e**, **g** The effect of serum- or LPA-induced cilia disassembly in STC1(**e**) or ODC1 (**g**) knockdown cells. RPE-1 cells were transfected and treated as described in Fig. 2b and 2c. **f**, **h** qPCR analysis confirms the knockdown efficiency of *STC1* (**f**) and *ODC1* (**h**) in (**e**) and (**g**), respectively. Source data are provided as a Source Data file. Three experiments were repeated independently with similar results in (**b**) and (**d**). Data are presented as mean ± S.D. of three independent experiments in (**a**), (**c**) and (**e-h**). ***P < 0.001. Two-tailed Student's t-test in (**f**) and (**h**); one way ANOVA test was performed followed by Bonferroni's multiple comparisons in in (**c**); two way ANOVA test was performed followed by Dunnett's multiple comparisons in (**a**).

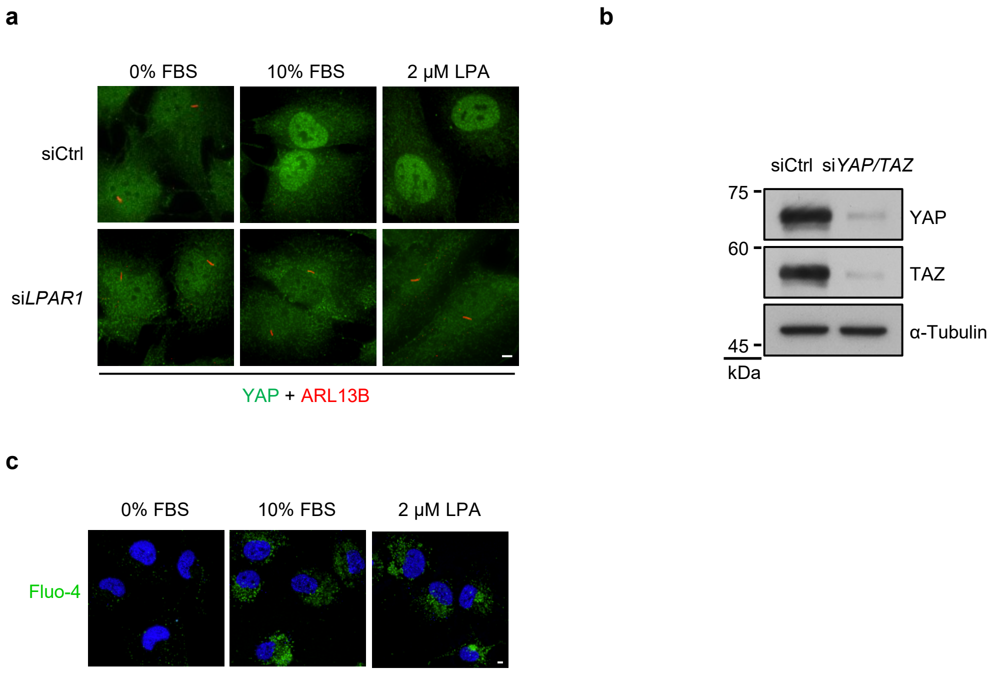

Supplementary Fig. 4 LPA activates YAP/TAZ and Ca^{2+} signaling. **a** Immunofluorescence images showing the localization of YAP in control or LPAR1-depleted RPE-1 cells. Ciliated RPE-1 cells were stimulated with 10% FBS or 2 μM LPA for 18h, and then cells were stained with anti-YAP (green) and anti-ARL13B (red) antibodies. Scale bar, 5 μm. **b** Immunoblots of RPE-1 cell lysates in Fig. 4c with the indicated antibodies. **c** Immunofluorescence images showing the activation of Ca^{2+} signaling in RPE1 cells after LPA treatment. Cells were starved for 48 h, and then treated with 10% FBS or 2 μM LPA for 10 min. Ca^{2+} signaling staining was performed using the Fluo-4, a calcium indicator. Scale bar, 5 μm. Three experiments were repeated independently with similar results in (**a**), (**b**) and (**c**).

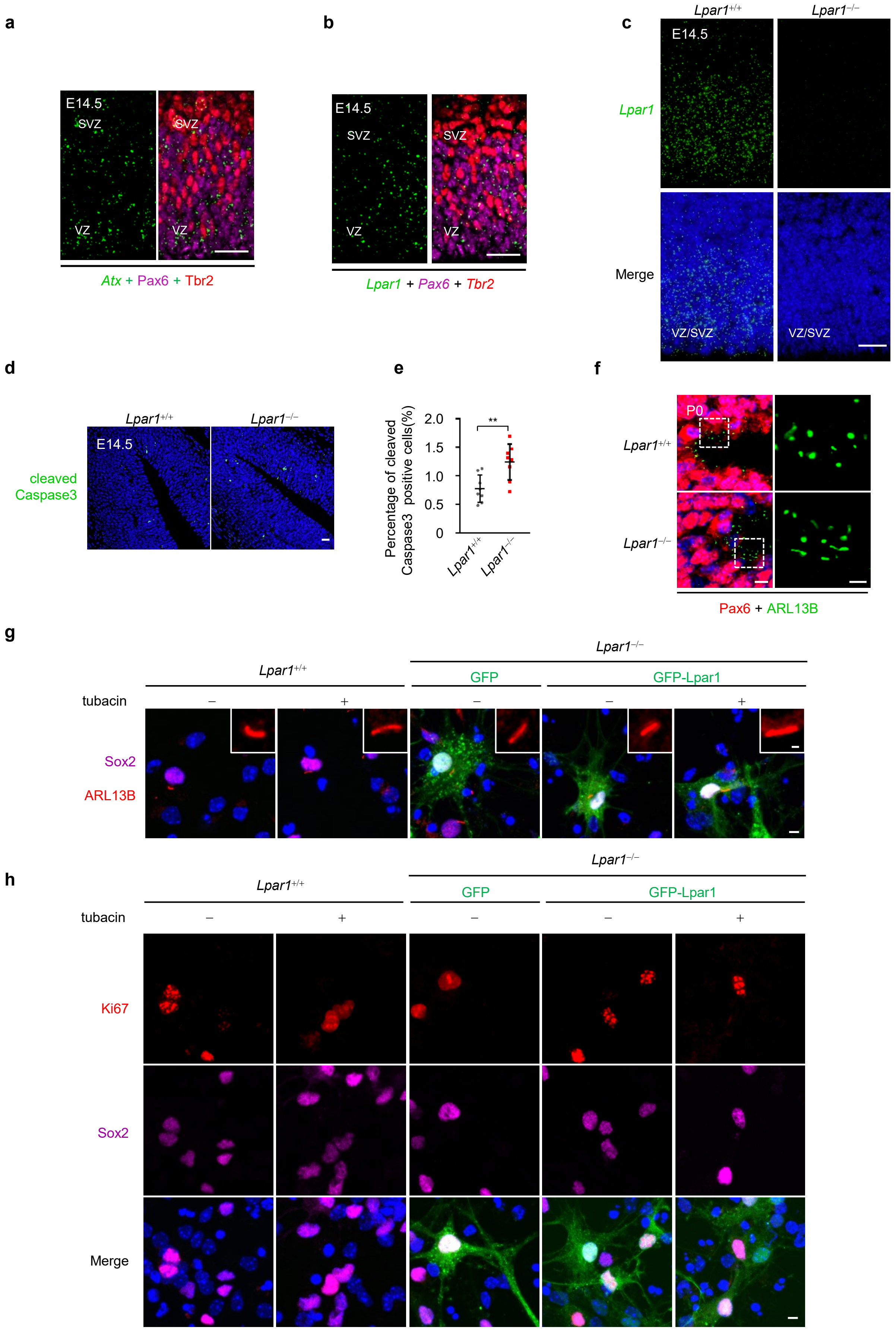

a
E14.5
SVZ
VZ
Atx + Pax6 + Tbr2
b
E14.5
SVZ
VZ
Lpar1 + Pax6 + Tbr2
c
Lpar1+/+
Lpar1−/−
E14.5
Lpar1
Merge
VZ/SVZ
d
Lpar1+/+
Lpar1−/−
E14.5
cleaved Caspase3
e
Percentage of cleaved Caspase3 positive cells(%)
2.0
1.5
1.0
0.5
0
**
Lpar1+/+
Lpar1−/−
f
P0
Lpar1+/+
Lpar1−/−
Pax6 + ARL13B
g
Lpar1−/−
Lpar1+/+
GFP
GFP-Lpar1
tubacin
−
+
Sox2
ARL13B
h
Lpar1−/−
Lpar1+/+
GFP
GFP-Lpar1
tubacin
−
+
Ki67
Sox2
Merge

Supplementary Fig. 5 Lpar1 modulates ciliogenesis and neurogenesis during cortical development. **a, b** RNAscope fluorescent in situ hybridization of *Atx* (**a**) and *Lpar1* (**b**) in wild-type mouse cortex. Cortices were stained with *Atx* mRNA puncta (green), anti-Pax6 (magenta) and anti-Tbr2 (red) antibodies (**a**) or *Lpar1* mRNA puncta (green), anti-Pax6 (magenta) and anti-Tbr2 (red) antibodies (**b**), respectively. Scale bar, 20 μm. **c** RNAscope fluorescent in situ hybridization of *Lpar1* in cortex with $Lpar1^{+/+}$ and $Lpar1^{-/-}$ mice at E14.5, *Lpar1* mRNA puncta (green), DNA (blue). Scale bar, 20μm. **d**, **e** VZ/SVZ cells in $Lpar1^{-/-}$ mice exhibit slightly increased apoptosis index. (**d**) Representative images of apoptosis cells. Cerebral cortices were stained with cleaved Caspase3 (green) and DNA (blue) in E14.5 $Lpar1^{+/+}$ and $Lpar1^{-/-}$ mice. Scale bar, 20 μm. (**e**) The percentage of cleaved Caspase3-positive cells from VZ/SVZ cells in (**d**), n = 8 sections from 4 mice. **f** Immunofluorescence images showing the elongated cilia in $Lpar1^{-/-}$ RG cells. $Lpar1^{+/+}$ and $Lpar1^{-/-}$ cortical sections at P0 were stained with cilia marker (ARL13B, green) and RG cells Pax6 (red). Scale bar, 5 μm (main image) and 1 μm (magnified region). **g** Representative images in Fig. 6g. Isolated cells from $Lpar1^{+/+}$ and $Lpar1^{-/-}$ cortices were stained with anti-Sox2 (magenta) and anti-ARL13B (red) antibodies. Scale bar, 5 μm (main image) and 1 μm (magnified region). **h** Representative images in Fig. 6h. Isolated cells from $Lpar1^{+/+}$ and $Lpar1^{-/-}$ cortices were stained with anti-Sox2 (magenta) and anti-Ki67 (red) antibodies. Scale bar, 5 μm (main image) and 1 μm (magnified region). Source data are provided as a Source Data file. Four experiments were repeated independently with similar results in (**a**), (**b**), (**c**), (**d**), (**f**) and (**h**). Data are presented as mean ± S.D. in (**e**). $^{**}P < 0.01$. Two-tailed Student's *t*-test in (**e**).
