# Supplementary material for: LPA signaling acts as a cell-extrinsic mechanism to initiate cilia disassembly and promote neurogenesis
Source: Nat Commun. 2021 Jan 28;12:662. doi: 10.1038/s41467-021-20986-y (PMC7843646; doi:10.1038/s41467-021-20986-y)
Supplement: Supplementary file 2 — Reporting Summary [file 41467_2021_20986_MOESM2_ESM.pdf]

nature research

Corresponding author(s): Hiui-Yan Li

Last updated by author(s): Nov 12, 2020

Reporting Summary

Nature Research wishes to improve the reproducibility of the work that we publish. This form provides structure for consistency and transparency in reporting. For further information on Nature Research policies, see our Editorial Policies and the Editorial Policy Checklist.

Statistics

For all statistical analyses, confirm that the following items are present in the figure legend, table legend, main text, or Methods section.

n/a	Confirmed	
☐	☒	The exact sample size (*n*) for each experimental group/condition, given as a discrete number and unit of measurement
☐	☒	A statement on whether measurements were taken from distinct samples or whether the same sample was measured repeatedly
☐	☒	The statistical test(s) used AND whether they are one- or two-sided<br>*Only common tests should be described solely by name; describe more complex techniques in the Methods section.*
☒	☐	A description of all covariates tested
☐	☒	A description of any assumptions or corrections, such as tests of normality and adjustment for multiple comparisons
☐	☒	A full description of the statistical parameters including central tendency (e.g. means) or other basic estimates (e.g. regression coefficient) AND variation (e.g. standard deviation) or associated estimates of uncertainty (e.g. confidence intervals)
☐	☒	For null hypothesis testing, the test statistic (e.g. *F*, *t*, *r*) with confidence intervals, effect sizes, degrees of freedom and *P* value noted<br>*Give P values as exact values whenever suitable.*
☒	☐	For Bayesian analysis, information on the choice of priors and Markov chain Monte Carlo settings
☒	☐	For hierarchical and complex designs, identification of the appropriate level for tests and full reporting of outcomes
☒	☐	Estimates of effect sizes (e.g. Cohen's *d*, Pearson's *r*), indicating how they were calculated

Our web collection on statistics for biologists contains articles on many of the points above.

Software and code

Policy information about availability of computer code

Data collection	For data acquisition on a DeltaVision Image Restoration Microscope, we used GE healthcare SoftWoRx 6.5.2.<br>For data collection on Zeiss LSM 880 microscope, we used ZEN 2.1 SP2 Black version 13.0.2.518 (ZEISS).
Data analysis	For image analysis, we used Volocity 6.0 software. Data acquisition and graphs were done in excel files and statistical analysis were done using SPSS 21.0 software. Sequenced reads of RNA-seq were aligned to hg38 with STAR 2.6.0a.

For manuscripts utilizing custom algorithms or software that are central to the research but not yet described in published literature, software must be made available to editors and reviewers. We strongly encourage code deposition in a community repository (e.g. GitHub). See the Nature Research guidelines for submitting code & software for further information.

Data

Policy information about availability of data

All manuscripts must include a data availability statement. This statement should provide the following information, where applicable:

- Accession codes, unique identifiers, or web links for publicly available datasets
- A list of figures that have associated raw data
- A description of any restrictions on data availability

All RNA sequencing files were deposited in the short read sequence archive (http://www.ncbi.nlm.nih.gov/sra) under BioProject ID PRJNA678549. Differentially expressed genes were mapped to the Kyoto Encyclopaedia of Genes and Genomes (KEGG) database (https://www.kegg.jp/) for pathway analysis. The authors declare that all data supporting the findings of this study are available within the article and its Supplementary Information files, or from the corresponding authors upon reasonable request.

Field-specific reporting

Please select the one below that is the best fit for your research. If you are not sure, read the appropriate sections before making your selection.

[x] Life sciences [ ] Behavioural & social sciences [ ] Ecological, evolutionary & environmental sciences

For a reference copy of the document with all sections, see nature.com/documents/nr-reporting-summary-flat.pdf

Life sciences study design

All studies must disclose on these points even when the disclosure is negative.

Sample size	No statistical methods were used to predetermine sample size. For all the experiments, we followed the routine practice in the similar studying fields. And it has been our routine practice to conduct experiments upon a relatively large but reasonable size. Results repeated at least three times were highly significant and consistent.
Data exclusions	No data were excluded from the analyses.
Replication	Attempts at replication were successful. For all experiments, at least 3 times independent experiments were repeated with the similar results as presented in this study. Detail information was involved in the Methods/Statistics and reproducibility section, as well as Statistics Source Data.
Randomization	Microscopic images were acquired randomly.
Blinding	The investigators were blinded to group allocation during data collection and analysis.

Reporting for specific materials, systems and methods

We require information from authors about some types of materials, experimental systems and methods used in many studies. Here, indicate whether each material, system or method listed is relevant to your study. If you are not sure if a list item applies to your research, read the appropriate section before selecting a response.

Materials & experimental systems

n/a	Involved in the study	
[ ]	[x]	Antibodies
[ ]	[x]	Eukaryotic cell lines
[x]	[ ]	Palaeontology and archaeology
[ ]	[x]	Animals and other organisms
[x]	[ ]	Human research participants
[x]	[ ]	Clinical data
[x]	[ ]	Dual use research of concern

Methods

n/a	Involved in the study	
[x]	[ ]	ChIP-seq
[x]	[ ]	Flow cytometry
[x]	[ ]	MRI-based neuroimaging

Antibodies

Antibodies used	Antibodies used in this study included mouse anti-Ac-tubulin antibody (1:800, T6793, Sigma, Clone-6-11B-1 lot: 017M4806V), rabbit anti-γ-tubulin antibody (1:600, T6557, Sigma, lot: 088M4786V), mouse anti-LPAR1 antibody (1:100, sc-515665, Santa,lot: F1616), mouse anti-α-tubulin (1:5000, T5168, Sigma,lot: 035M4878V), mouse anti-Flag antibody (1:1000, F3165, Sigma,lot: 080M6034), mouse anti-Gα 12 antibody (1:200, sc-515445, Santa, lot: A1416), mouse anti-Gα 13 antibody (1:200, sc-293424, Santa, lot: B0216), mouse anti-Gα q antibody (1:200, sc-136181, Santa, lot: K1115), mouse anti-Gα 11 antibody (1:200, sc-390382, Santa, lot: G2017), mouse anti-YAP antibody (1:1000, sc-101199, Santa, lot: F0316), rabbit anti-TAZ antibody (1:4000, 66500-1-Ig, Proteintech, lot: 10005041), rabbit anti-Aurora A antibody (1:1000, 4718s, Cell Signaling Technologies, lot: 5), rabbit Anti-HDAC6 antibody (1:1000, 7558T, Cell Signaling Technologies, lot: 1), rabbit anti-ARL13B antibody (1:400, 17711-1-AP, Proteintech, lot: 00066216), mouse anti-BrdU antibody (1:250, 11-286-C100, Exbio, lot: 534848) , rabbit anti-Pax6 antibody (1:500, 901301, Biolegend, lot: B244513), rat anti-Tbr2 antibody (1:500, 14-4875-82, Thermo Fisher Scientific, lot: 2009821), rabbit anti-Satb2 antibody (1:500, ab92446, Abcam, lot: GR3206907-1), mouse anti-Tbr1 antibody (1:250, 66564-1-Ig, Proteintech, lot:10005277), rabbit anti-p-H3(Ser10) antibody (1:500, 9701s, Cell Signaling Technologies, lot: 2297281), rabbit anti-Phospho-Aurora A (Thr288) antibody (1:100, MA5-14904, Thermo Fisher Scientific, lot: SC2358571H), goat anti-Sox2 antibody (1:300, Sc-17320 , Santa, lot: I1015), rabbit anti-Ki67 antibody (1:500, 9129T, Cell Signaling Technologies, lot: 3), rabbit anti-cleaved Caspase3 antibody (1:400, 9664, Cell Signaling Technologies, lot: 4), mouse anti-Flag-M2 FITC (1:8000, F4049, Sigma,lot: SLBV8285), Goat anti-mouse Alexa Fluor 488 (1:500, A11029, Thermo Fisher Scientific, lot: 1874804), Goat anti-mouse Alexa Fluor 546 (1:500, A11030,Thermo Fisher Scientific, lot: 1829584), Goat anti-mouse Alexa Fluor 647 (1:500, A21235, Thermo Fisher Scientific, lot: 2088736), Goat anti-Rabbit Alexa Fluor 488 (1:500, A11034, Thermo Fisher Scientific, lot: 1885241), Goat anti-Rabbit Alexa Fluor 546 (1:500, A11035, Thermo Fisher Scientific, lot: 1904467), Goat anti-Rabbit Alexa Fluor 647 (1:500, A21245, Thermo Fisher Scientific, lot: 2051068), Goat anti-Rat Alexa Fluor 555 (1:500, A21434, Thermo Fisher Scientific, lot: 1670155), Goat anti-Rat Alexa Fluor 488 (1:500, A11006, Thermo Fisher Scientific, lot: 1689880), Donkey anti-goat Alexa Fluor 647 (1:500, A21447, Thermo Fisher Scientific, lot: 1841382).

Validation	Anti-Ac-tubulin antibody was validated by the manufacturer using western blot. Species reactivity: mouse, rat, chicken, protista, bovine, invertebrates, human, monkey, pig, frog, plant and hamster. Anti-γ-Tubulin antibody was validated by the manufacturer using western blot. Species reactivity: bovine, mouse, rat, chicken, Xenopus, human, hamster, canine. Anti-LPAR1 antibody was validated by the manufacturer using western blot. Species reactivity: mouse, rat and human. Anti-α-Tubulin was validated by the manufacturer using western blot. Species reactivity: chicken, kangaroo rat, sea urchin, rat, Chlamydomonas, bovine, human, African green monkey and mouse. Anti-Flag antibody was validated by the manufacturer using western blot. Species reactivity: all. Anti-Gα 12 antibody was validated by the manufacturer using western blot. Species reactivity: mouse, rat and human. Anti-Gα 13 antibody was validated by the manufacturer using western blot. Species reactivity: mouse, rat and human. Anti-Gα q antibody was validated by the manufacturer using western blot. Species reactivity: mouse, rat, canine and human. Anti-Gα 11 antibody was validated by the manufacturer using western blot. Species reactivity: mouse, rat and human. Anti-YAP antibody was validated by the manufacturer using western blot. Species reactivity: mouse, rat and human. Anti-TAZ antibody was validated by the manufacturer using western blot. Species reactivity: mouse, rat and human. Anti-Aurora A antibody was validated by the manufacturer using western blot. Species reactivity: human and monkey. Anti-HDAC6 antibody was validated by the manufacturer using western blot. Species reactivity: human and monkey. Anti-ARL13B antibody was validated by the manufacturer using western blot. Species reactivity: human, mouse, rat and dog. Anti-BrdU antibody was validated by the manufacturer using FC. Species reactivity: all. Anti-Pax6 antibody was validated by the manufacturer using western blot. Species reactivity: human, mouse and rat. Anti-Tbr2 antibody was validated by the manufacturer using IHC. Species reactivity: mouse. Anti-Satb2 antibody was validated by the manufacturer using western blot. Species reactivity: human, mouse and rat. Anti-Tbr1 antibody was validated by the manufacturer using western blot. Species reactivity: human, rat, mouse and pig. Anti-Ctip2 antibody was validated by the manufacturer using western blot. Species reactivity: human, rat and mouse. Anti-p-H3(Ser10) antibody was validated by the manufacturer using western blot. Species reactivity: human, mouse, rat, monkey, drosophila and yeast. Anti-Phospho-Aurora A (Thr288) antibody was validated by the manufacturer using IF. Species reactivity: human. Anti-Sox2 antibody was validated by the manufacturer using IF. Species reactivity: human, mouse and rat. Anti-Ki67 antibody was validated by the manufacturer using IF. Species reactivity: human, mouse and rat. Anti-cleaved Caspase3 antibody was validated by the manufacturer using western blot. Species reactivity: human, mouse, rat and monkey. Anti-Flag-M2 FITC was validated by the manufacturer using IF. Species reactivity: all. Anti-mouse Alexa Fluor 488 was validated by the manufacturer using IF. Species reactivity: mouse.Anti-mouse Alexa Fluor 546 was validated by the manufacturer using IF. Species reactivity: mouse. Anti-mouse Alexa Fluor 647 was validated by the manufacturer using IF. Species reactivity: mouse. Anti-Rabbit Alexa Fluor 488 was validated by the manufacturer using IF. Species reactivity: rabbit. Anti-Rabbit Alexa Fluor 546 was validated by the manufacturer using IF. Species reactivity: rabbit. Anti-Rabbit Alexa Fluor 647 was validated by the manufacturer using IF. Species reactivity: rabbit. Anti-Rat Alexa Fluor 555 was validated by the manufacturer using IF. Species reactivity: rat. Anti-Rat Alexa Fluor 488 was validated by the manufacturer using IF. Species reactivity: rat. Anti-goat Alexa Fluor 647 was validated by the manufacturer using IF. Species reactivity: goat.

Eukaryotic cell lines

Policy information about cell lines

Cell line source(s)	hRPE-1 cells and IMCD-3 were kindly provided by profferesor Xueliang Zhu (Shanghai Institute of Biochemistry and Cell Biology, CAS). HEK293T cells were obtained from the ATCC (CRL-3216)
Authentication	The cell lines were not authenticated.
Mycoplasma contamination	We confirm that all cell lines have been tested negative for mycoplasma contamination.
Commonly misidentified lines (See ICLAC register)	No common misidentified cell lines were used

Animals and other organisms

Policy information about studies involving animals; ARRIVE guidelines recommended for reporting animal research

Laboratory animals	The animals we used in this study were mouse C57BL/6J. Mice were kept under specific-pathogen-free conditions, with controlled temperature (20–25 °C), humidity (40–60%) and light cycle (12 h light/dark). All embryos used for this study were obtained from natural mattings of virgin females 8-12 weeks old.
Wild animals	The study did not involve wild animals, no animals in the study were collected from the field.
Field-collected samples	The study did not involve wild animals, no animals in the study were collected from the field.
Ethics oversight	All animal experiments were performed with the approval of the Institutional Animal Care and Use Committee of Academy of Military Medical Sciences (IACUC-DWZX-2019-506).

Note that full information on the approval of the study protocol must also be provided in the manuscript.
