# Supplementary material for: LPA signaling acts as a cell-extrinsic mechanism to initiate cilia disassembly and promote neurogenesis
Source: Nat Commun. 2021 Jan 28;12:662. doi: 10.1038/s41467-021-20986-y (PMC7843646; doi:10.1038/s41467-021-20986-y)
Supplement: Supplementary file 3 — Source Data [file 41467_2021_20986_MOESM3_ESM.zip › Souse Data of Western blot .pptx]

## Slide 1
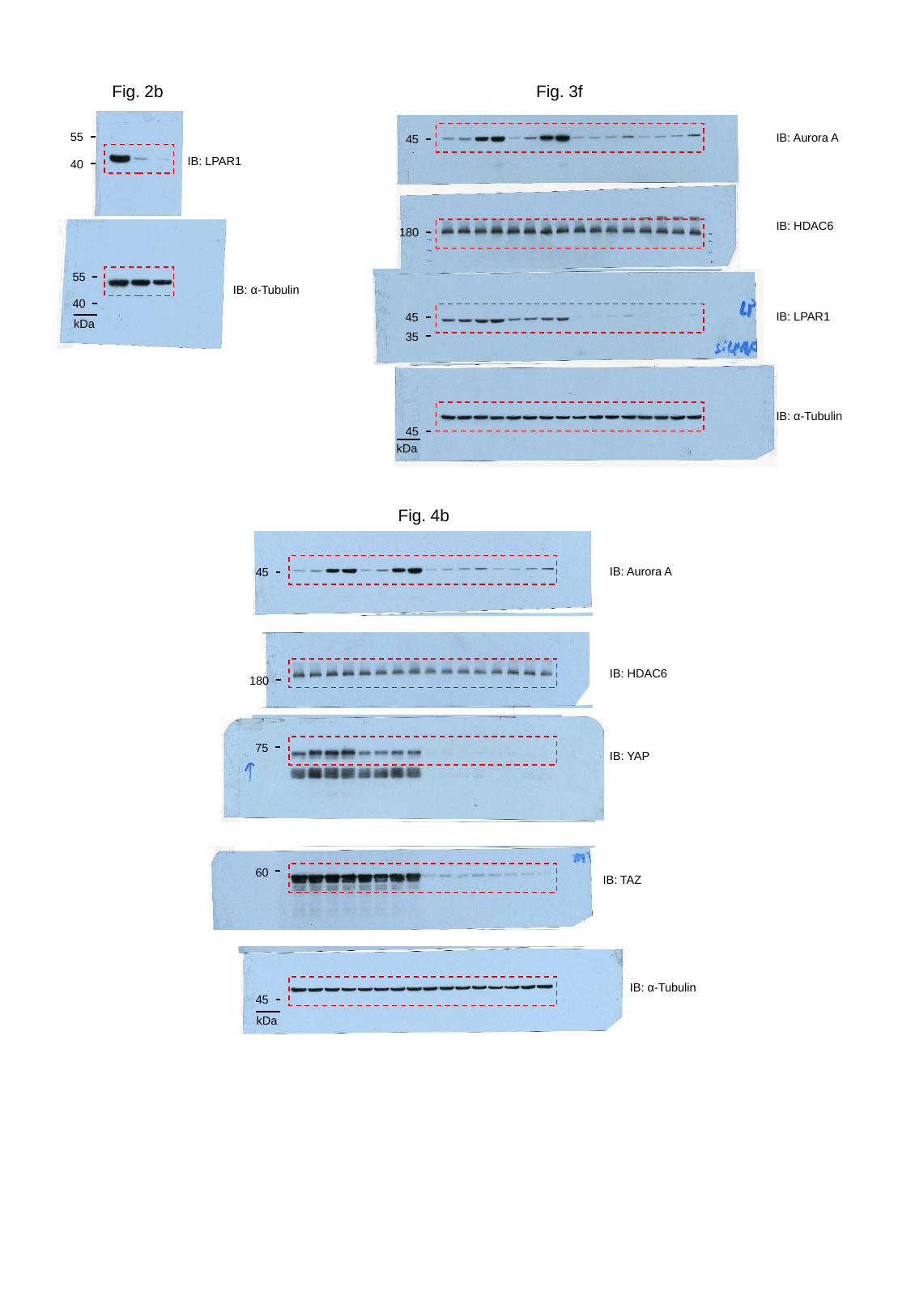

Fig. 2b
Fig. 3f
55
IB: Aurora A
45
IB: LPAR1
40
IB: HDAC6
180
55
IB: α-Tubulin
40
IB: LPAR1
45
kDa
35
IB: α-Tubulin
45
kDa
Fig. 4b
IB: Aurora A
45
IB: HDAC6
180
75
IB: YAP
60
IB: TAZ
IB: α-Tubulin
45
kDa

## Slide 2
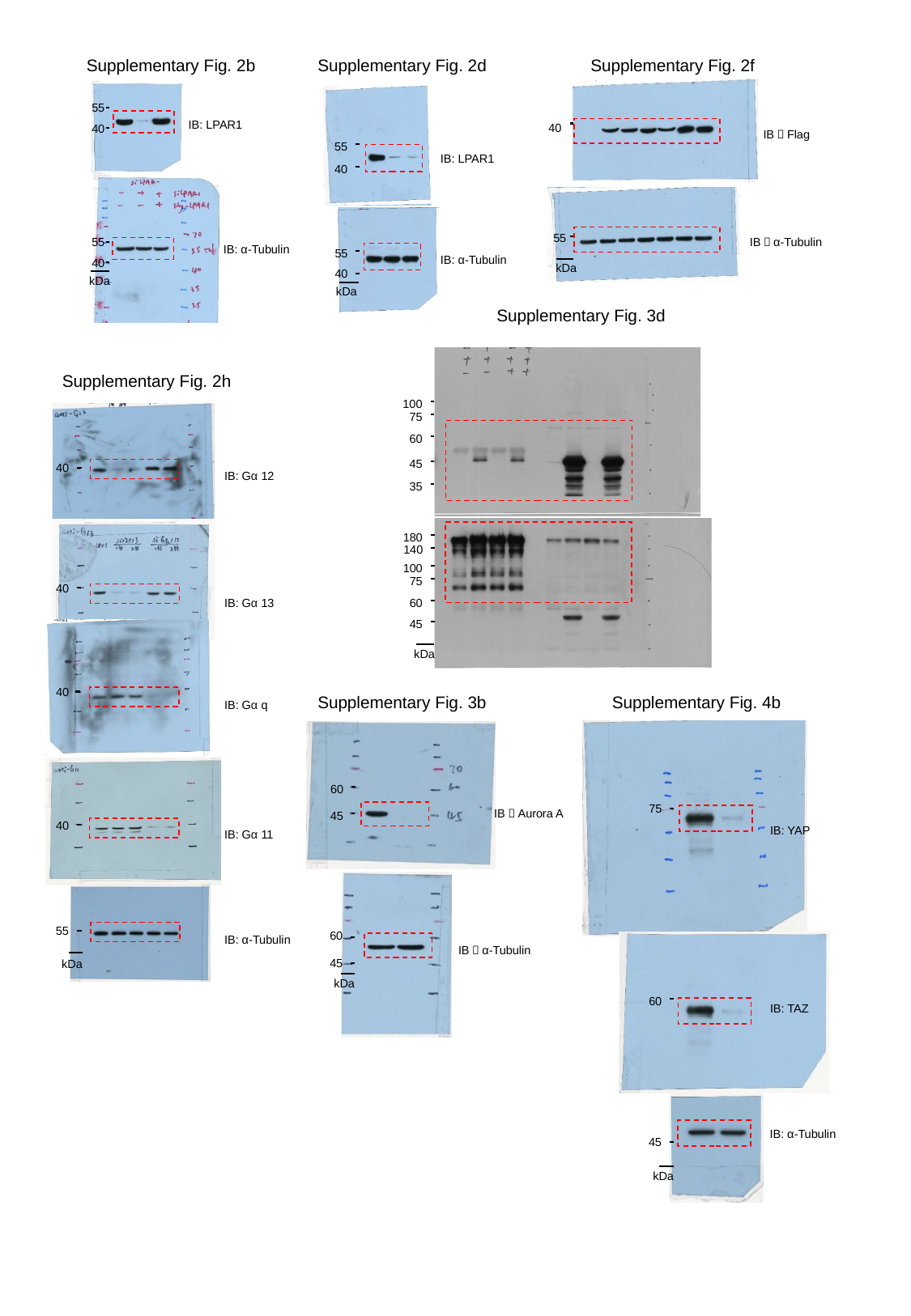

Supplementary Fig. 2d
55
IB: LPAR1
40
55
IB: α-Tubulin
40
kDa
Supplementary Fig. 2f
Supplementary Fig. 2b
55
IB: LPAR1
40
55
40
kDa
40
IB：Flag
55
IB：α-Tubulin
kDa
IB: α-Tubulin
Supplementary Fig. 3d
100
75
60
45
IB: HA
35
180
140
IB: Flag
100
75
60
45
kDa
Supplementary Fig. 2h
40
IB: Gα 12
40
IB: Gα 13
40
Supplementary Fig. 3b
Supplementary Fig. 4b
IB: Gα q
75
IB: YAP
60
IB: TAZ
IB: α-Tubulin
45
kDa
60
IB：Aurora A
45
60
IB：α-Tubulin
45
kDa
40
IB: Gα 11
55
IB: α-Tubulin
kDa
